# ShinyDataMatcher: A user-friendly application for integrating survey data

**DOI:** 10.1371/journal.pone.0353530

**Published:** 2026-07-14

**Authors:** Lucia Guastadisegni, Fedele Greco, Carlo Trivisano

**Affiliations:** 1 Department of Statistics, University Carlos III of Madrid, Getafe, Madrid, Spain; 2 Department of Statistical Sciences “Paolo Fortunati”, University of Bologna, Bologna, Italy; Utrecht University: Universiteit Utrecht, NETHERLANDS, KINGDOM OF THE

## Abstract

In this work, we introduce ShinyDataMatcher, a user-friendly R Shiny application designed to support the integration of survey data through statistical matching. The tool enables practitioners to import, explore and process survey data, harmonize variables, select appropriate matching variables, and apply a wide range of macro- and micro-level matching methods without writing any code. The application guides the user through the full workflow of a matching exercise, from data preparation to the creation of a synthetic matched dataset, and includes diagnostic tools for assessing matching quality. To illustrate its capabilities, we present an application based on the Italian Household Budget Survey (HBS) and the Survey on Household Income and Wealth (SHIW), where the goal is to fuse income and expenditure information and to construct a Social Accounting Matrix (SAM). The example also highlights how repeated random hot-deck imputations can be used to account for the additional uncertainty induced by statistical matching. Overall, ShinyDataMatcher provides a transparent and accessible environment for exploring, prototyping, and implementing statistical matching procedures, lowering the technical barriers that often limit their use in applied and official-statistics contexts.

## Introduction

Statistical matching is a set of techniques used to integrate information from surveys that refer to different statistical units, in which a subset of variables is jointly observed while other variables are observed separately [1–3]. The need to combine information from different surveys arises because official statistical studies often focus on specific aspects of households or individuals. However, many research questions require the joint analysis of variables that are not collected within a single data source. For instance, the study of economic well-being typically relies on information on income, wealth, and consumption [4]; however, in many countries these three dimensions are not jointly measured in a single survey. Statistical matching makes it possible to integrate multiple surveys to obtain complete datasets for such analyses (see for example [5–9]). A classic further example is the construction of Social Accounting Matrices (SAM), which require linking information from different sources to provide a coherent representation of economic and social flows [3,10].

Typically, statistical matching involves two data sources, and the matching relies on variables observed in both surveys [11,12]. Depending on the purpose of the study, matching can be conducted at either the macro or micro level. The macro approach aims to estimate features of the joint distribution of variables that are not jointly observed, whereas the micro approach aims to create a synthetic file in which each statistical unit is equipped with all relevant variables, even if they originate from separate data sources.

A standard assumption ensuring model identifiability in both macro and micro approaches is the Conditional Independence Assumption (CIA), which states that variables observed separately are conditionally independent given the common variables [2,13,14]. Under the CIA, both parametric and non-parametric statistical matching methods can be implemented effectively.

Among parametric macro methods, classical approaches rely on maximum likelihood (ML) estimation under the assumption of multivariate normality [15]. Other procedures include least squares estimators for regression parameters [16] and estimators based on observed sample counterparts [17]. For categorical variables, ML estimation of multinomial parameters is typically employed. Non-parametric macro methods include the estimation of empirical cumulative distribution functions and kernel density estimators.

For micro-level matching under the CIA, parametric approaches include conditional mean matching and its stochastic variants, which reduce to regression imputation under multivariate normality [18]. Non-parametric micro methods consist of hot-deck procedures [19–22], such as random, rank, or distance-based matching. Mixed approaches combine parametric estimation with hot-deck assignment, such as predictive mean matching [23,24]. More recent methods relax the CIA by leveraging auxiliary information [22,25]. Comprehensive reviews of approaches with and without auxiliary information can be found in [26].

Although several of these methods have been implemented in STATA [27] and R (via the StatMatch package; [28]), their practical application remains challenging. Before matching can be carried out, users must perform multiple preprocessing steps: importing data, aligning statistical units, harmonizing variables, and selecting appropriate matching variables [29]. The harmonization of common variables may require recoding categories, creating new comparable variables, and ensuring high data quality [30]. The selection of matching variables is crucial, particularly under CIA, and variables should be strong predictors of the target variables [5].

Depending on the nature of the variables involved, an appropriate matching method should then be selected, and the quality of the resulting matched data must be evaluated. These operations require programming skills and a detailed knowledge of statistical software, which may be a barrier for many practitioners.

To address these challenges, we developed ShinyDataMatcher, an R Shiny application that supports all phases of statistical matching, from data import and harmonization to the selection of matching variables, the implementation of various macro and micro methods, and the download of the matched dataset, without requiring users to write code. The application is provided through the ShinyStatMatcher R package, available on GitHub. The package can be installed in R using: remotes::install_github(“fedele-greco/ShinyStatMatcher”). The package exposes a single function, run_ShinyStatMatcher(), which launches the application.

Interactive tools such as ShinyDataMatcher can play a key role in making complex statistical methods accessible to a broader community of practitioners. To show the use of ShinyDataMatcher, we also present an example of how to create a matched dataset through the application based on two surveys: the Household Budget Survey (HBS; [31]) and the Survey on Household Income and Wealth (SHIW; [32]). Using the matched dataset, we construct a module of the Social Accounting Matrix (SAM) related to households and assess the additional variability introduced by the matching procedure.

This article is organized as follows: the section *Statistical Framework and Methods* provides a description of statistical matching techniques at both the macro and micro levels under the CIA. The section *Statistical Matching through ShinyDataMatcher* presents the application features used to perform all steps of statistical matching. The section *Real Data Application* illustrates a real data application using ShinyDataMatcher, concerning the construction of the SAM matrix. Finally, the section *Conclusions* offers concluding remarks and discussion.

## Statistical framework and methods

**Fig 1:**
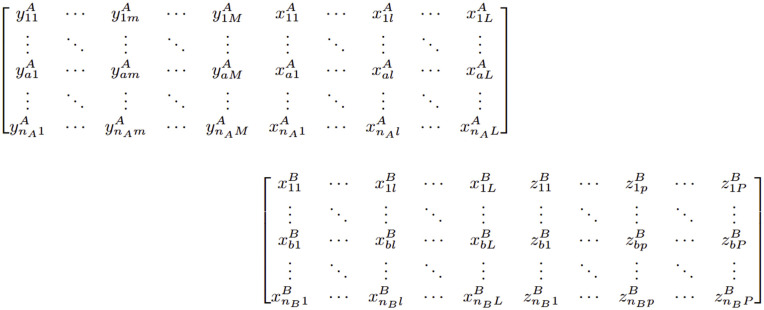
Survey information.

Let $𝐘=(Y_{1},\cdots ,Y_{M}{)}^{⊤}$, $𝐙=(Z_{1},\cdots ,Z_{P}{)}^{⊤}$ and $𝐗=(X_{1},\cdots ,X_{L}{)}^{⊤}$ be vectors of random variables (r.v.s) of dimension *M*, *P* and *L*, respectively. Consider two datasets *A* and *B* referring to $n_{A}$ and $n_{B}$ statistical units, respectively. For each unit, independent and identically distributed (i.i.d.) observations generated from the joint distribution f(x,y,z) are available. The units in *A* have Z missing, hence the data matrix gathered from *A* can be denoted as $\left[{𝐗}^{A}\;\;{𝐘}^{A}\right]$, where ${𝐗}^{A}\in {\mathbb{R}}^{n_{A}\times L}$ and ${𝐘}^{A}\in {\mathbb{R}}^{n_{A}\times M}$, respectively. Conversely, the units in *B* have Y missing and the data matrix is $\left[{𝐗}^{B}\;\;{𝐙}^{B}\right]$, where ${𝐗}^{B}\in {\mathbb{R}}^{n_{B}\times L}$ and ${𝐙}^{B}\in {\mathbb{R}}^{n_{B}\times P}$, respectively. Fig 1 displays the overlapping and non-overlapping information gathered by the two surveys:

The macro approach aims to estimate some summary feature of the joint distribution f(x,y,z), while the micro approach aims to construct a new synthetic dataset in which the missing variables in the recipient survey are imputed. In a statistical matching framework, samples *A* and *B* are assumed to be drawn from the same target population. Moreover, it is assumed that A∪B can be considered as a unique sample of size $n_{A}+n_{B}$ and that, from such a sample, a synthetic dataset can be derived in which the missing data mechanism is either missing completely at random (MCAR) or missing at random (MAR), see [3,12,15,26].

A common assumption that guarantees model identifiability is the *Conditional Independence Assumption* (CIA) between Y and Z given X, that is [3]:


$$\begin{array}{c} f(x,y,z)=f_{Y|X}(y|x)\cdot f_{Z|X}(z|x)\cdot f_{X}(x). \end{array}$$ (1)

The CIA cannot be tested from the data; this has generated several concerns and discussion on the implications of its violation. Nonetheless, matching methods under the CIA are very popular and statistical software for their implementation is available, a prominent example being the R package StatMatch [28]. In what follows, we sketch the main idea of these methods, both at the macro and micro levels, as they are implemented in the ShinyDataMatcher application. Technical details about the methods are provided in S1 Appendix. In particular, we consider *M* = *P* = 1, which is the case implemented in the application: hence, *y* and *z* are scalar variables, while **x** denotes an *L*-dimensional random vector.

### Parametric methods

Parametric methods assume a parametric form for the joint distribution in (1), that is


$$\begin{array}{c} f(𝐱,y,z;\theta )=f_{Y|X}(y|𝐱;\theta_{y|𝐱})\cdot f_{Z|𝐗}(z|𝐱;\theta_{z|𝐱})\cdot f_{𝐗}(𝐱;\theta_{𝐱}), \end{array}$$ (2)

where $\theta_{y|𝐱}\in \Theta_{y|𝐱}$, $\theta_{z|𝐱}\in \Theta_{z|𝐱}$, $\theta_{𝐱}\in \Theta_{𝐱}$. The macro approach include the estimation of the parameter in $\Theta_{y|𝐱},\Theta_{z|𝐱}$ and $\Theta_{𝐱}$, that depends on the nature of **X**, *Y* and *Z*. Details on the macro methods for both continuous and categorical variables are reported in S1 Appendix, section S1.1.

For the micro methods, in the case *Y* and *Z* are continuous, a complete synthetic dataset can be obtained by estimating the regression model of *Y* on **X** from dataset *A* and the regression model of *Z* on **X** from dataset *B*. We consider the imputation of the missing variable *Z* in dataset *A*; however, the same theory can also be applied to the imputation of the missing variables *Y* in dataset *B*. The imputation can then be performed using two parametric methods: conditional mean matching, where each missing value is replaced with the expected value of the missing variable, and stochastic regression imputation, where random values are added to the prediction to account for the variability of the imputed values around their conditional mean. Details on the two methods are provided in S1 Appendix, section S1.2.

### Non-parametric methods

To avoid misleading results due to incorrect parametric assumptions, several non-parametric methods have been proposed for statistical matching at both the micro and macro levels, although the latter has received less attention. The main methods implemented in ShinyDataMatcher for micro-level matching applications are listed below. Non-parametric methods at the micro level are referred to as hot-deck methods [22]. In general, for all micro-level methods, dataset *A* serves as the recipient file, while dataset *B* is the donor file. Missing values for the variable Z in the recipient dataset are filled with observed values from the donor dataset, rather than with predicted values as in parametric micro methods. Typically, $n_{B}\geq n_{A}$. The final matched synthetic dataset consists of $n_{A}$ units and includes the values x, y, and $\tilde{z}$, where $\tilde{z}$ has been imputed. The main techniques applied under the CIA are listed below. First, there is the random hot-deck method, which randomly selects a donor record from the donor file for each record in the recipient file. Another method is the rank hot-deck method [21], where an ordinal matching variable *X* is used to select donors for assignment to records in dataset *A*. [19] proposed the distance hot-deck method, in which each unit in the recipient dataset is matched with the closest donor in terms of a chosen distance metric. Distance-based hot-deck techniques are flexible, as they can accommodate various distance measures. Further details on the above methods are provided in S1 Appendix, section S2.

## Statistical matching through ShinyDataMatcher

**Fig 2:**
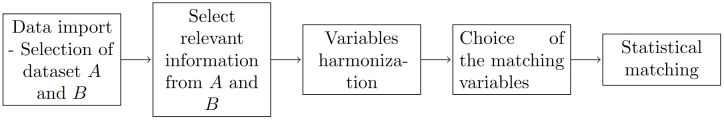
Workflow of statistical matching.

The methods described in the *Statistical Framework and Methods* section assume that datasets *A* and *B* are integrable, meaning they use consistent units, time periods, and variable definitions. In practice, however, this assumption rarely holds. Recorded variables often differ, for example in how categories are defined and coded for categorical variables or in the choice of measurement units for quantitative variables. As a result, users often need to devote considerable effort to harmonizing the datasets. This process can be time consuming, depending on the analyst’s expertise, and is susceptible to multiple sources of error. Once data harmonization has been completed, the next step is the careful selection of matching variables, which requires the application of appropriate selection techniques. The matching method should then be chosen according to the specific characteristics of each variable involved. This entire process calls for programming skills and familiarity with statistical software such as R: this is an expertise that many practitioners may lack. In this section, we sketch the phases of the statistical matching process, by highlighting the role of ShinyDataMatcher and its potential in practical work settings. To this aim, we consider applications where dataset *A* plays the role of the recipient, while *B* is the donor. In Fig 2 the workflow of statistical matching is displayed, which can be performed using ShinyDataMatcher.

Each phase of the statistical matching workflow is structured into distinct tabs within the application ShinyDataMatcher, developed using R Shiny. The current version supports statistical matching with univariate *Y* and *Z* variables. Each tab’s content is discussed in detail in the following subsections.

### Data import/selection of dataset A and B

The goal of the initial phase of statistical matching is to work with two datasets, *A* and *B*, ideally aligned in terms of reference periods and unit definitions [33]. Achieving these two objectives is challenging in practice, as for example harmonizing the reference periods, and users often make assumptions to apply statistical matching methods [3].

**Fig 3:**
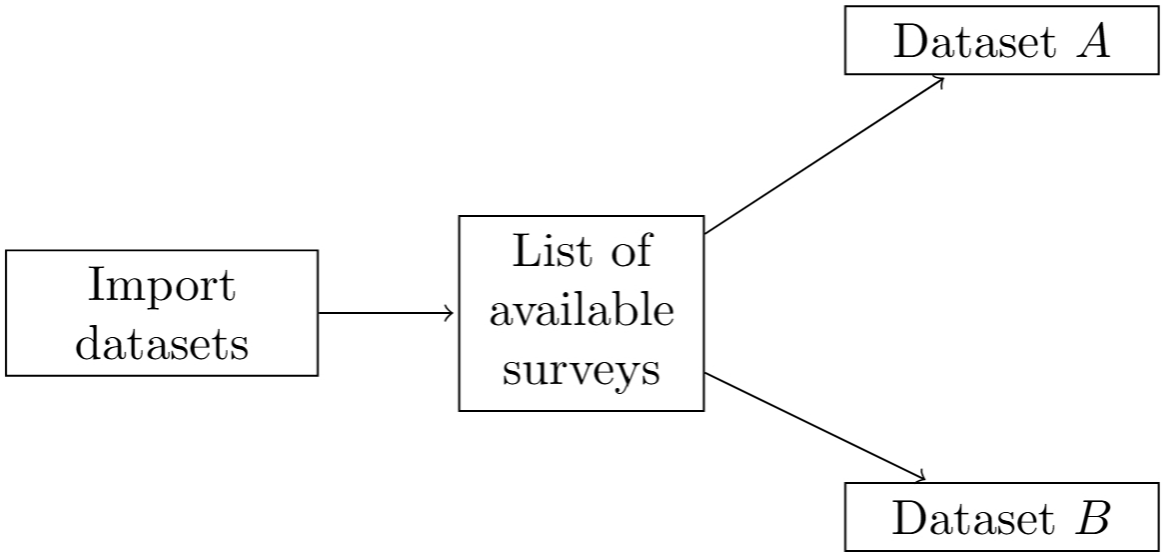
Data import and dataset selection.

The application allows users to perform the steps illustrated in Fig 3.

More specifically, the first tab of the application provides the following features:

- Data Import: The app allows to import rectangular data files, where rows correspond to observational units (e.g., individuals, households, firms), while columns correspond to variables or attributes describing each unit. Data import is performed leveraging the rio::import function of the rio R package [34], which supports several formats such as .csv, .txt, .xlsx, among others. Once a file is uploaded, data are added to the following list of available surveys.
- List of Available Surveys: This list contains all the datasets that can be managed in the app. By default, this list contains a subset of the HBS and SHIW data corresponding to the year 2020, that are used for illustrative purposes in the Real Data Application Section.
- Dataset *A*/*B* Selection: Users can choose Dataset *A* (recipient) and Dataset *B* (donor) from the list of available surveys. If a ID variable containing unit identifiers is included in the dataset, it can be selected in this tab. If no ID variable is selected, the app automatically generates one for subsequent analyses.

### Select relevant information from A and B

**Fig 4:**
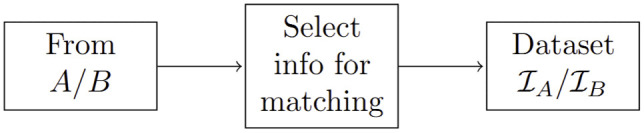
Select information for matching.

Survey datasets typically contain a large number of raw variables that are not necessarily relevant for the matching application at hand. Since both datasets may contain numerous variables, users are allowed to select only the raw variables needed for matching, which we refer to as information. The resulting reduced datasets are denoted as ${ℐ}_{A}$ and ${ℐ}_{B}$. In this tab, users can perform the steps reported in Fig 4.

### Variables harmonization

This phase requires appropriate transformations of raw variables selected at the former step (${ℐ}_{A}$ and ${ℐ}_{B}$) in order to create harmonized datasets that we denote as ${𝐗}_{A}$ and ${𝐗}_{B}$. Such datasets include candidate matching variables that have been harmonized and the target variables. The aim is to address any inconsistencies among similar variables so they can be used in the statistical matching procedure. In the app, the variable harmonization consists of two tabs where donor and recipient are managed separately. They both include the same features, described below. The tool currently allows one transformation at a time. From the dataset ${ℐ}_{A}$ (or ${ℐ}_{B}$), each variable can be selected. Transformations can be applied either to multiple selected raw variables or to a single variable. If only one variable is selected, the following transformations can be applied:

- Rename a variable: This makes it possible to assign a more informative name to a variable and, more importantly, to harmonize variable names between the two datasets for subsequent use as a matching variable in the matching algorithm.
- Quantitative to Categorical: This allows to transform a quantitative variable into a categorical one. Users can select the number of categories and define the cut-off points for grouping as well as labels assigned to each category.
- Recode a Factor: This allows to recode the categories of a categorical variable.
- Add as it is: This allows the user to simply add a variable to the dataset $X_{A}$ (or $X_{B}$) without applying any transformation.

It could also be useful to create new variables that summarize information from a set of variables in the two datasets. When multiple variables are selected in ${ℐ}_{A}$ (or in ${ℐ}_{B}$), they can be transformed by means of row-wise operations:

- Count the Number of Occurrences: Given a set of variables, it is possible to create a new variable that counts how many times a specific category (or categories) appears within those variables.
- Check for Occurrences of Categories Row-Wise: When multiple variables are selected, another option is to create a binary variable that indicates whether a specific category appears in a series of variables.
- Sum Row-Wise: for numerical variables, this allows to compute row-wise sums.

**Fig 5:**
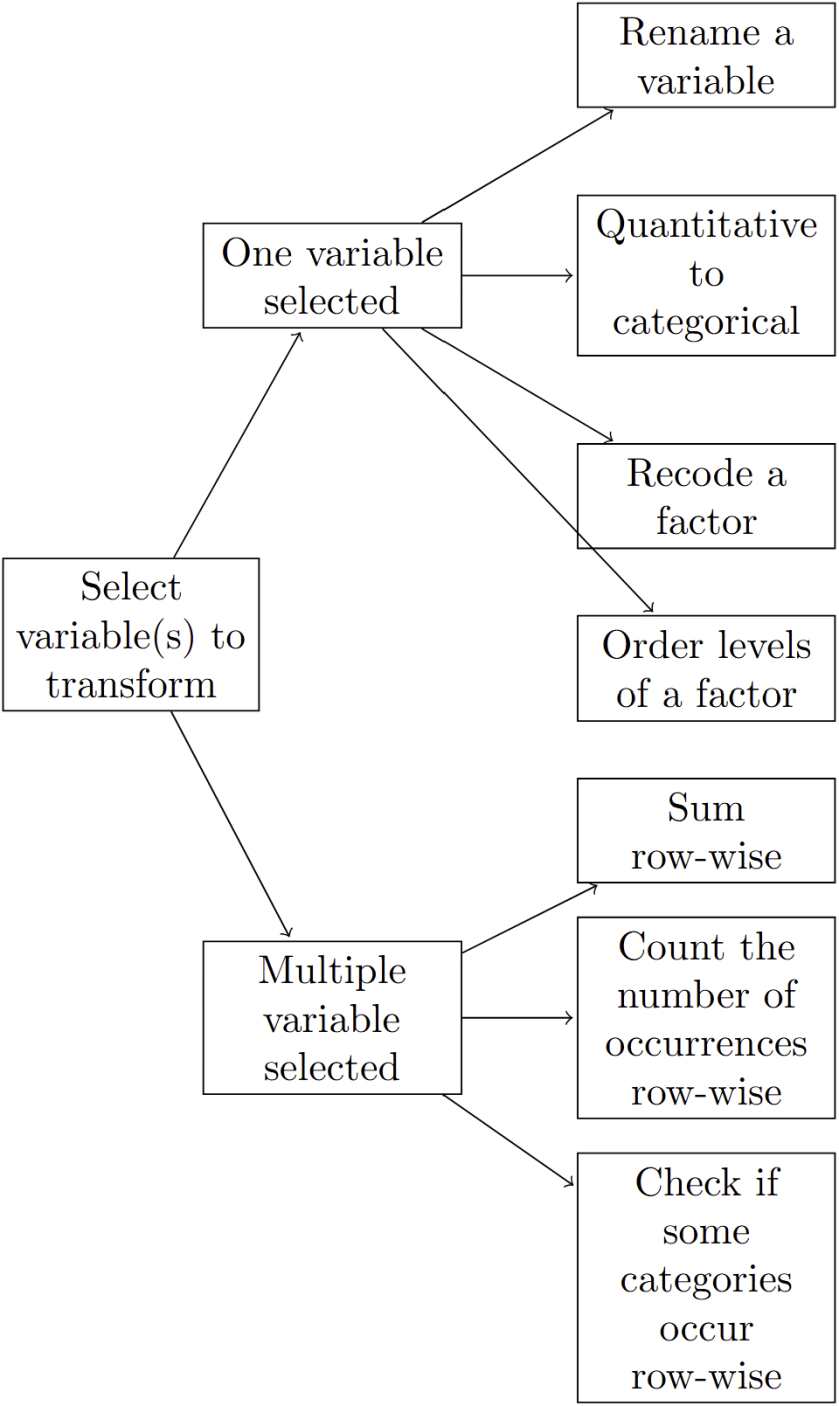
Variable harmonization.

These variables can be stored either as numeric or as factors. Fig 5 summarizes the above transformations:

At the end of this phase, the user has selected non-overlapping variables *Y* and *Z* along with candidate matching variables, creating two datasets ${𝐗}_{A}$ and ${𝐗}_{B}$. The user has the possibility to check if the harmonization process has been accomplished by employing the Check harmonization tool which allows to check if variables share the same name, if they are of the same type and if they share the same categories in the case of categorical variables.

### Choice of the matching variables

**Fig 6:**
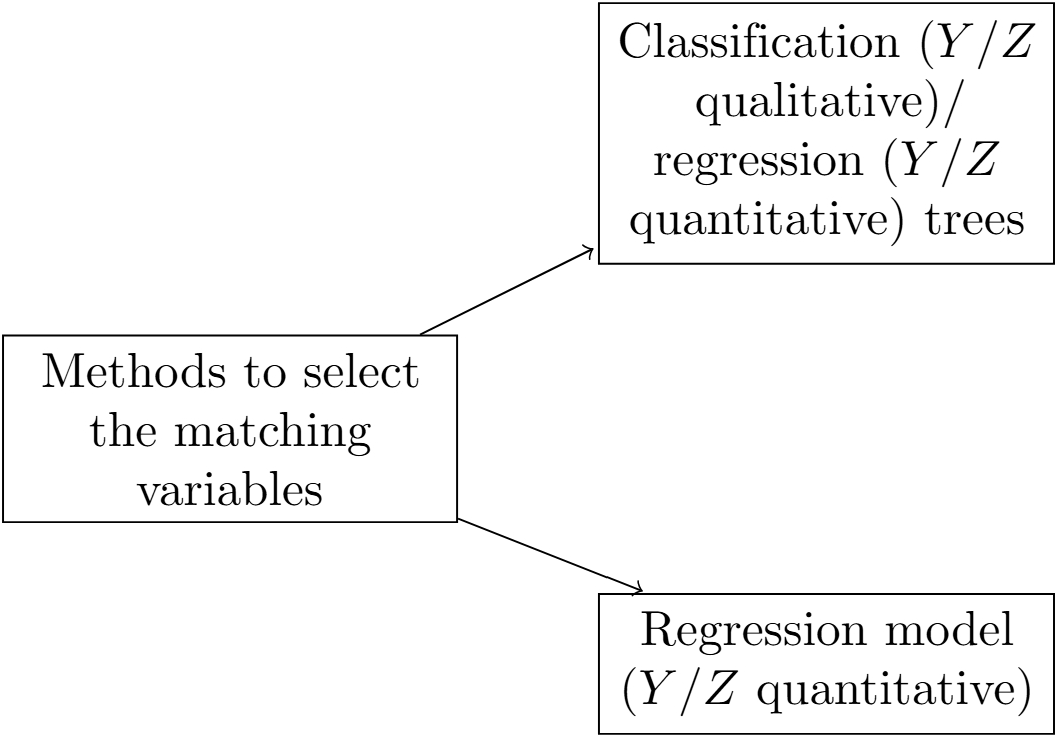
Selection of matching variables.

Variables in ${𝐗}_{A}$ and ${𝐗}_{B}$ are only candidates to be used as matching variables. In this phase, one aims to identify variables that are statistically related to either Y or Z [22,35]. To this aim, ShinyDataMatcher includes a tab for analyzing both the donor and recipient data. Focusing on the donor, just as an example, consider variables *Z* and ${𝐗}_{B}$. If *Z* is continuous, two methods can be used to select the matching variables: the linear regression model or the regression tree. Matching variables are identified as those that have a significant effect on *Z* in the regression model and those utilized in the regression tree algorithm. Additionally, a plot of the regression tree is provided for better visualization. If *Z* is categorical, classification trees can be used to identify matching variables. The tab’s features are summarized in Fig 6.

### Statistical matching

Depending on the objective of the study, macro or micro methods can be applied to perform statistical matching, according to the methods described in section *Statistical Framework and Methods*.

**Fig 7:**
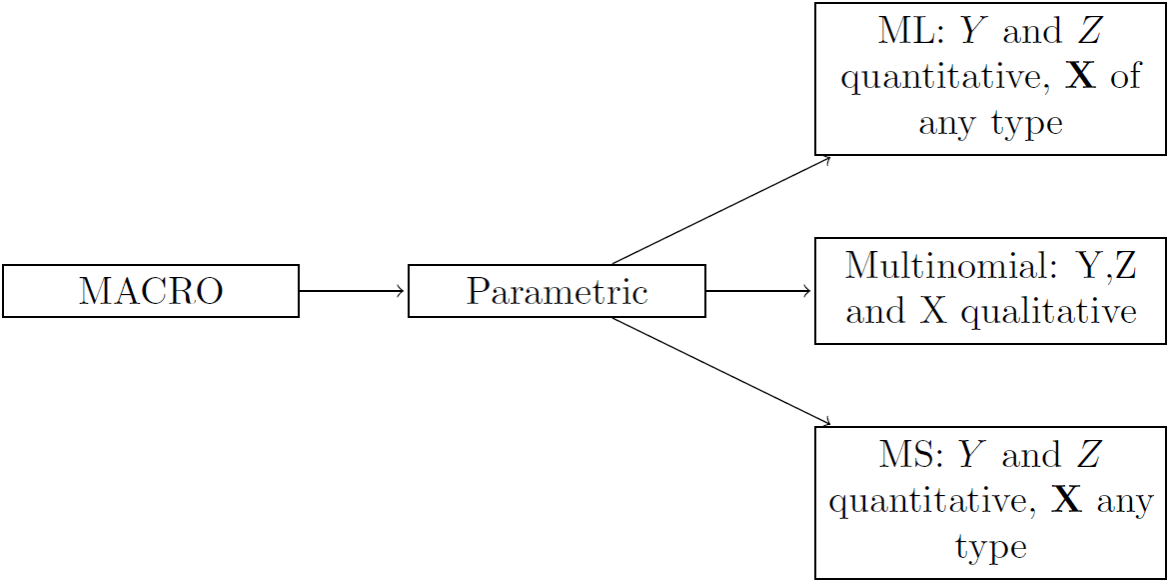
Statistical macro matching methods.

The last two tabs of the application allow users to perform statistical matching at the macro or micro level respectively. When the objective is macro-level matching, first, the user selects the variable *Y* from dataset *A*, the variable *Z* from dataset *B*, and the matching variables **X**. The macro parametric methods are made available in the application through a selection menu. Depending on the nature of *Z* and *Y*, and in some cases X, specific macro methods can be selected, as shown in Fig 7.

**Fig 8:**
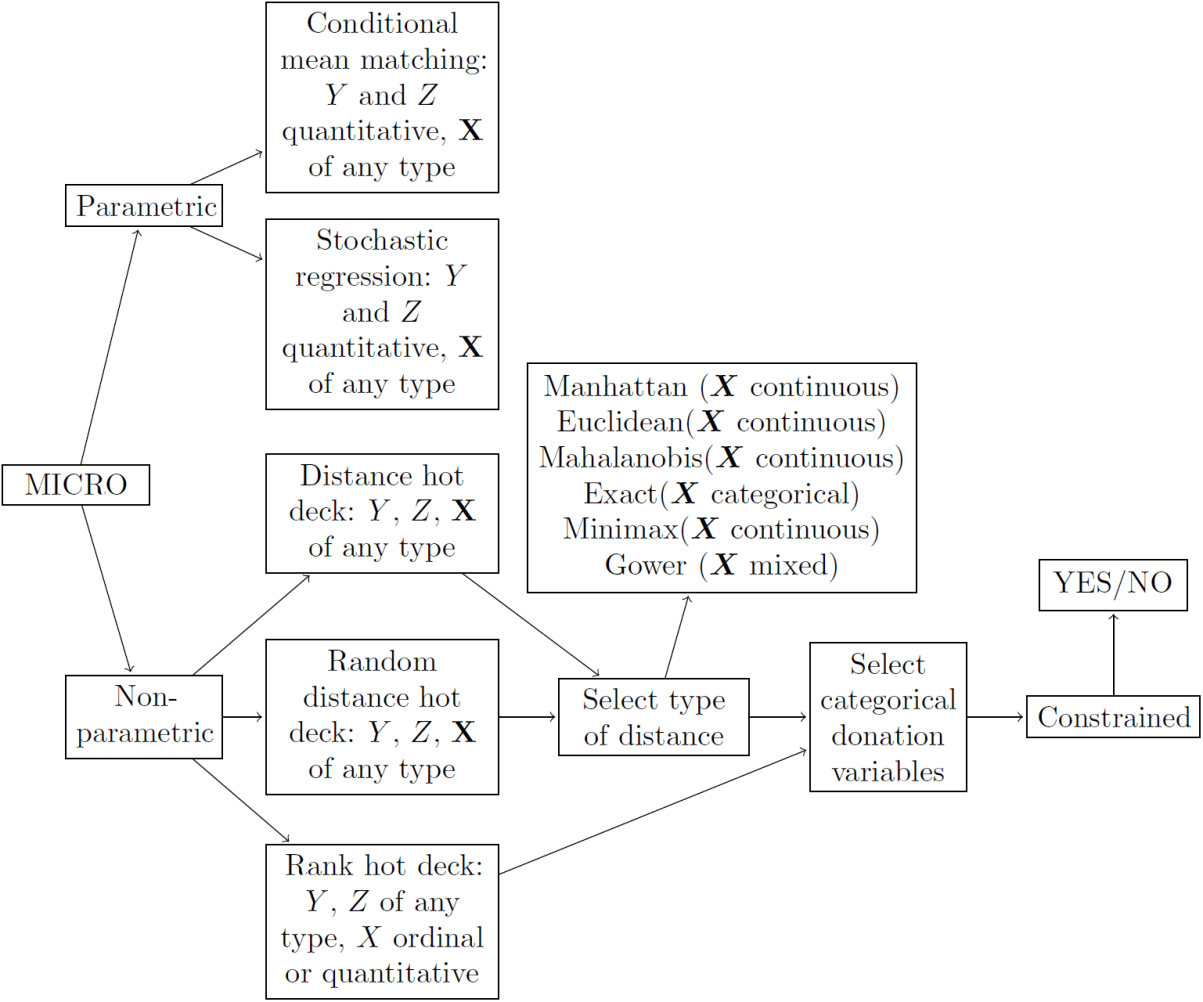
Statistical micro matching methods.

When the objective is micro-level matching, all the possible choices, which depend on the nature of *Y*, *Z*, and X, as well as on the selected methods, are summarized in Fig 8.

When using a random method, the user can choose to generate more than one matched variable. This is particularly useful when one wants to evaluate uncertainty due to matching in the application at hand: this idea is developed in the *Real Data Application* section. Once the chosen algorithm has been run, the variable *Z* is imputed to each statistical unit in dataset *A*. Some evaluation methods for the matching process have been developed and made available in the Summaries tab, which displays basic summary statistics and plots for the matched variables. Users can download the matched dataset in the Storage and download tab. To demonstrate the functionality of the app, a real-data example illustrating the use of each tab is presented in the following section.

## Real data application

This section illustrates the use of the app in a real case study.

We consider a subset of the Households Budget Survey [31] and Survey on Household Income and Wealth [32] datasets. The application aims: (i) to replicate the application presented in [3] through ShinyDataMatcher, and (ii) to construct a module of the Social Accounting Matrix (SAM) related to households using matched data, while also accounting for the additional variance of the estimates due to the matching procedure. [3] considered the construction of the Social Accounting Matrix using the SHIW and HBS datasets for the year 2000. We consider more recent data, namely those for the year 2020. A Social Accounting Matrix (SAM) is a structured data system that records economic and social flows in a matrix layout. It incorporates indicators such as per-capita income, growth, and the income and spending patterns of various economic actors, classified by key characteristics. Its main roles are to summarize a country’s economic and social structure for a given year and to supply the data foundation for models that describe the economy at a point in time and evaluate the effects of policy changes [3]. Specifically, the SAM module related to households arranges household categories in the rows, while income and expenditure variables are represented in the columns. Detailed income variables are available in SHIW, whereas expenditure categories are available in HBS. To this aim, we start by developing a micro-matching application with the aim to impute the total households expenditure variable (*Z*) in the SHIW file, which provides only information concerning household income (*Y*). Hence in the application SHIW plays the role of the recipient, while HBS is the donor.

The analysis, performed with the application, is summarized in the following steps:

### Data import/delection of dataset A and B

In the tab Available Surveys, we select SHIW as the recipient and HBS as the donor from the list of pre-loaded datasets available in ShinyDataMatcher. The rationale is that the HBS includes more units than the SHIW and is therefore typically selected as the donor.

To proceed, in the field Choose dataset A (the recipient) we select SHIW-2020 and set the ID variable to NQUEST, which identifies the statistical units. In the field Choose dataset B (the donor) we select HBS-2020. Since no ID variable is contained in the survey, the ID variable is left empty and is automatically generated by the application. Users can explore the dataset, including the number of units and available variables, and visualize summary statistics and univariate plots. S1 and S2 Figs report screenshots of this tab, displaying some content for both the considered datasets.

### Select relevant information for matching

In general, users should select raw variables that carry the same information across the two datasets, such as demographic variables (e.g., household size, age, sex, education, occupation) and wealth indicators (e.g., home ownership, dwelling surface area, second home ownership). We retained only a subset of the raw variables that share common characteristics in SHIW and HBS, reported in [36], in order to simplify the application, since the main objective of this section is to illustrate the app’s functionality.

The raw variables to be selected for matching in SHIW are reported in Table 1.

**Table 1:** Raw variables for matching dataset A.

Variable	Description
Y	Net disposable income
STUDIO_1_Fact to STUDIO_9_Fact	Educational qualification of each member
B01_1_Fact to B01_9_Fact	Employed
APQUAL2_1_Fact to APQUAL2_9_Fact	Professional position of each member
AREA5_1_Fact	Geographic area of residence
SUPAB	What is the surface area (in square meters) of this dwelling/apartment?
NCOMP	Number of people living in this household, 0 years or older, as of 31-12-2020

A more detailed description of these variables is given in S1 Table. In practice, this selection is carried out through the Select Info for Matching – Recipient Data tab. In the Select information from the recipient survey section, the user should click on the variables listed in Table 1. If the variables are selected correctly, they will appear under Selected information from the recipient survey (${ℐ}_{A}$).

The raw variables to be selected for matching in HBS are reported in the Table 2.

**Table 2:** Raw variables for matching dataset B.

Variable	Description
sp_tot_str_aggr_1	Total Household Expenditure
c_titstu_1_Fact to c_titstu_12_Fact	Education level of each component
cond_1_Fact to cond_12_Fact	Condition of each component
rip_Fact	Geographical region (5 level)
c_superf	Area of the dwelling
c_ncmp_fatto	Total number of members in the cohabiting family

A more detailed description of these variables is given in S2 Table. The same process outlined for SHIW should be followed for HBS by using the Select Info for Matching – Donor Data tab, in order to create the dataset ${ℐ}_{B}$. The selection process is depicted in S3 and S4 Figs, which show screenshots of the Select info for matching tab: on the left, the selectable information is displayed, while on the right, the selected information is shown. The output of this phase is then used in the subsequent phase devoted to variable harmonization.

### Variables harmonization

For Datasets A and B, a series of transformations is carried out to construct two datasets, $X_{A}$ and $X_{B}$, each comprising a set of harmonized variables, along with the target variable *Y* in Dataset A and *Z* in Dataset B. In all subsequent phases, users should note that the app is case-sensitive (e.g., Area ≠ AREA).

First, we summarize the transformations required for Dataset A, performed through the tab Data wrangling – Recipient Data. The variable Y is simply added to the dataset $X_{A}$ through the transformation Add as it is. To save the variable into $X_{A}$, the user has to click the Click when done button (this final step must be performed for all transformed variables described in this section). AREA5_1_Fact is transformed using the Recode a Factor function. The categories are recoded as follows: North-East as NE, North-West as NW, Centre as C, South as S, and Islands as I. Through the New variable name field, the variable is renamed Area. This harmonization process is illustrated in S5 Fig. The set of variables STUDIO_1_Fact–STUDIO_9_Fact contributes to the creation of several derived variables. First, all variables in this set are selected, and the transformation Count the number of occurrences is applied. The first variable created is named through the New variable name field No_tit, indicating the number of members with no school qualifications in the household. In the field used to select the modality (Select categories), the value none is specified. The resulting variable must be saved as numeric. Then, STUDIO_1_Fact–STUDIO_9_Fact are selected again and, following the same procedure outlined above, the variable Comp, representing members with up to 8 years of schooling, is created. In this case, in the field used to select the modality, the values elementary school certificate and lower middle school certificate are specified. Similarly, the variable Diploma, corresponding to individuals with 9–13 years of education, is created. In the field used to select the modality, the values professional diploma (3 years) and high school diploma are specified. Finally, the variable Degree, indicating members holding a university degree, is created. In the field used to select the modality, the values university diploma/bachelor’s degree, master’s degree, and postgraduate specialization are specified. All these variables should be saved as Numeric.

Labour market status is similarly constructed. The variables B01_1_Fact–B01_9_Fact are selected and the transformation Count the number of occurrences is applied. Through the New variable name field, the new variable is named Job, indicating the number of household members with a job. In the field used to select the modality, the value Yes is specified. The resulting variable must be saved as numeric. This process is illustrated in S9 Fig. To compute the variable representing the number of retired members, the variables APQUAL2_1_Fact–APQUAL2_9_Fact are selected and the transformation Count the number of occurrences is applied. The new variable is named Ret through the appropriate field. In the field used to select the modality, the values Retired (work-related pension) and Retired (non-work-related: disability/survivor/social pension) are specified. Also in this case the variable is saved as numeric.

The variable SUPAB is renamed Housesup through the Rename a variable transformation.

Finally, the variable NCOMP is transformed through the Quantitative to Categorical transformation, to create a variable representing household size. In the New variable name field, the name NCOMP is specified. In the field Select the number of categories, the value 5 is entered. Then, the cut points are defined as follows: Cut 1 = 1, Cut 2 = 2, Cut 3 = 3, Cut 4 = 4. In the field Insert categories labels, the following labels are specified: Label for category 1 = 1, Label for category 2 = 2, Label for category 3 = 3, Label for category 4 = 4, and Label for category 5 = >=5. This harmonization process is illustrated in S7 Fig.

The harmonization process for Dataset B follows a similar strategy and is performed through the tab Data wrangling – Donor Data.

The variable sp_tot_str_aggr_1 is renamed as Z through the Rename a variable transformation.

The variable rip_Fact is transformed through the Recode a Factor function. The categories are recoded as follows: Nord-ovest as NW, Nord-est as NE, Centro as C, Sud as S, and Isole as I. Through the New variable name field, the variable is renamed Area. This process is illustrated in S6 Fig. The set of variables c_titstu_1_Fact–c_titstu_12_Fact contributes to the creation of several derived variables, all of which must be saved as numeric. First, all variables in this set are selected, and the transformation Count the number of occurrences is applied.

The first variable is created by specifying the name No_tit in the New variable name field. In the field used to select the modality, the value nessun titolo is specified. Then, with the same variables selected again and following the same procedure outlined above, the variable Comp is created. In the field used to select the modality, the values Elementari and Medie are specified. Similarly, the variable Diploma is created. In the field used to select the modality, the value superiori is specified. Finally, the variable Degree is created. In the field used to select the modality, the value Laurea e post-laurea is specified.

Labour market status is constructed from the variables cond_1_Fact–cond_12_Fact. These variables are selected and the transformation Count the number of occurrences is applied. Through the New variable name field, the new variable is named Job. In the field used to select the modality, the value Occupato/a is specified. The resulting variable must be saved as numeric. This process is illustrated in S10 Fig. To compute the variable representing the number of retired members, the same variables are selected and the transformation Count the number of occurrences is applied and the new variable is named Ret through the appropriate field. In the field used to select the modality, the value Persona ritirata dal lavoro is specified. Also in this case the variable is saved as numeric.

The variable c_superf is renamed Housesup through the Rename variable transformation.

Finally, the variable c_ncmp_fatto is transformed through the Quantitative to Categorical transformation. In the New variable name field, the name NCOMP is specified. In the field Select the number of categories, the value 5 is entered. Then, the cut points are defined as follows: Cut 1 = 1, Cut 2 = 2, Cut 3 = 3, Cut 4 = 4. Finally, in the field Insert categories labels, the following labels are specified: Label for category 1 = 1, Label for category 2 = 2, Label for category 3 = 3, Label for category 4 = 4, and Label for category 5 = >=5. This harmonization process is illustrated in S8 Fig. The transformations explained above required for Dataset *A* and *B* in the example at hand are summarized in S3 and S4 Tables, respectively.

The harmonization process presented above has been carried out following the approach presented in [3]. In general, for variables that are very similar, such as geographic area, harmonization is straightforward and can be achieved simply by renaming categories and variable names. For example, in the case of house area, the only discrepancy between the two datasets is the variable name. More challenging are variables that retain information at the individual level (e.g., educational attainment variables such as STUDIO_1-STUDIO_9 in SHIW and c_titstu_1_Fact-c_titstu_12_Fact in HBS). In these cases, to avoid losing information, it is important to construct new variables that summarize individual-level information and make it comparable at the household level. For this reason, the transformation Counting the number of occurrences has been chosen. Harmonizing the number of components variables by creating a categorical variable is also necessary in order to use them later as stratification variables in the analysis.

At the end of the harmonization phase, it is possible to check if the harmonization process has been accomplished by employing the Check harmonization tool which allows to check if variables share the same name, if they are of the same type and if they share the same categories in the case of categorical variables. S11 Fig reports the screenshot of such check after the harmonization performed for the application at hand.

### Choice of the matching variables

To choose the matching variables, we first analyze the results of a regression model on Dataset A using the tab Selection of matching variable – Recipient Data. The following options are selected: Select Response Variable = Y, Select Predictors = Area, No_tit, Comp, Diploma, Degree, Job, Ret, Housesup, NCOMP, and Select the method = Multivariate Regression. As shown in S12 Fig, the regression analysis on the SHIW dataset indicates that the variables with a significant effect on Y are Degree, Job, Area, and Housesup.

A similar analysis is performed for the variables in the Donor dataset using the tab Selection of matching variable – Donor Data. The following options are selected: Select Response Variable = Z, Select Predictors = Area, No_tit, Comp, Diploma, Degree, Job, Ret, Housesup, NCOMP, and Select the method = Multivariate Regression. The results, shown in S13 Fig, indicate that the variables with a statistically significant effect on Z are Diploma, Degree, Ret, Job, Area, and Housesup. At the end of the selection process, we chose to adopt as matching variables the number of household members with a degree (Degree), with a diploma (Diploma), with a job (Job), and those retired (Ret). Moreover, we considered the house surface area (Housesup). Following the approach and guidelines in [3], to define the donation classes, we use the geographic area (Area) and the number of household members grouped into five categories (NCOMP).

### Statistical matching

To import expenditure data from HBS into SHIW at the household level, we adopt the Random Distance Hot Deck method. The choice of this method is motivated by the fact that, by using a random approach, the user can generate more than one matched variable. This is important for evaluating uncertainty, as will be clarified in the next subsection.

Between the Random Hot Deck and the Random Distance Hot Deck methods, we choose the latter because it produces more accurate results. In particular, it does not assign donors completely at random to recipient units, as the matching is based on a distance metric. In detail, we use the tab Micro Matching and the section Matching. The following options are selected: Select Z from the donor = Z, Select matching variables = Degree, Diploma, Job, Ret, Housesup, Methods = Non-parametric: Random Distance Hot Deck, Donor classes = YES, Select donor classes = NCOMP, Area, and Select a distance = Manhattan. The use of the app’s interface for the effective implementation of matching methods is shown in S14 Fig. Since the method involves randomization, we assess the sensitivity of the final output, the SAM, to the specific random vector of imputed values by generating multiple replications of the algorithm, which lead to multiple generations of the matching variable Z. To do so, we set the option Number of matched variables to generate to 500 replications of Z, which in turn produce 500 SAMs. This computation requires several minutes. To reduce computational time, for the purpose of replicating the application, we recommend setting this number to a lower value (e.g., 10). Once the algorithm has been run, the evaluation of matching quality for each generated dataset can be inspected via the Evaluation of Matching menu. Finally, the Storage and Download menu allows exporting the matched data. In detail, users must first click on the Store Matched Variables button to save the matched variables, and then on the Download data button to download the original dataset (see S15 Fig), which consists of the SHIW dataset augmented with 500 vectors of Z expenditure data. Such an exported dataset will constitute the input for the construction of the SAMs.

### Building the SAM matrix

To illustrate a possible use of the matched data generated through the application, we construct a SAM module related to households. The household SAM module is a matrix that reports, for each household type, expenditures (broken down into a large number of categories) and incomes (employee income, self-employed income, interest, dividends, rents, social security transfers). Households can be classified in different ways, for example by area (region) of residence.

We consider a very small portion of the SAM matrix to highlight the drawbacks of statistical matching procedures. We report the average income and the average expenditure for Italian households classified by geographical area. To show the increase in variability introduced by the matching procedures, we split the SAM matrix into two smaller tables: the first refers to income, observed in SHIW, and the second to total expenditure, imputed in SHIW through the app as described in the previous section.

Income estimates are obtained using the sampling weights available in the SHIW database, which also includes jackknife replicate weights that account for the complexity of the sampling design and allow for consistent variance estimation through the jackknife method. The results for average income by geographical area are shown in Table 3.

**Table 3:** Average Income estimate ($\bar{Y}$), Variance of the estimate ($V(\bar{Y})$), coefficient of variation of the estimates ($cv(\bar{Y})$).

Area	$\bar{Y}$	$V(\bar{Y})$	$cv(\bar{Y})$
Center	39503	185612	0.011
Islands	28783	6347	0.003
North-East	45977	234472	0.011
North-West	49490	173524	0.008
South	26774	5369	0.003

As expected, the coefficients of variation are small, consistent with the fact that the parameters refer to estimation domains with large sample sizes. On the other hand, when estimating average expenditure, it should be noted that the estimates are obtained from matched data, and therefore the variance of the estimators should be inflated to account for the additional uncertainty. As shown in the previous section, users can generate, through the app, more than one replication of *Z*, following the multiple imputation method [37].

We adopt *K* = 500 expenditure vectors, denoted by $Z^{\left(k\right)},\;k=1,\ldots ,K$, to obtain, for each imputed vector, the estimate of the average expenditure ${\bar{Z}}^{\left(k\right)}$ and its variance estimate $\hat{V}({\bar{Z}}^{\left(k\right)})$. The multiple-imputation (MI) estimate is obtained by averaging the imputed values of *Z* over the replications [38] as


$$\begin{array}{c} \bar{Z}=\frac{1}{K}\sum_{k=1}^{K}{\bar{Z}}^{\left(k\right)}. \end{array}$$ (3)

The associated variance is given by [38]


$$\begin{array}{c} \hat{V}(\bar{Z})=W+\left(\frac{K+1}{K}\right)B, \end{array}$$ (4)

where *W* is the within-imputation variability


$$\begin{array}{c} W=\frac{1}{K}\sum_{k=1}^{K}\hat{V}\;\left({\bar{Z}}^{\left(k\right)}\right), \end{array}$$ (5)

and *B* is the between-imputation variability, which captures the heterogeneity of the estimates derived from the matching procedure


$$\begin{array}{c} B=\frac{1}{K-1}\sum_{k=1}^{K}{\left({\bar{Z}}^{\left(k\right)}-\bar{Z}\right)}^{2}. \end{array}$$ (6)

Results concerning the average expenditure estimates are reported in Table 4, where it can be seen that the most relevant source of uncertainty is associated with the matching procedure. This leads to a considerable inflation of the coefficient of variation compared with what one would obtain using a single vector of matched data, as commonly happens in practical applications where the additional variability due to the matching procedure is ignored.

**Table 4:** MI Expenditure estimate ($\bar{Z}$), Within-imputation variance (*W*), Between-imputation variance (*B*),coefficient of variation of the estimates ($cv(\bar{Z})$).

Area	$\bar{Z}$	*W*	*B*	$\hat{V}(\bar{Z})$	$cv(\bar{Z})$
Center	2624	34	2870	2910	0.021
Islands	2034	56	3907	3971	0.031
North-East	2854	105	3544	3656	0.021
North-West	2713	167	2752	2924	0.020
South	1873	64	1478	1545	0.021

The method described above to compute the variance of an estimate based on matched data has some limitations. Indeed, as observed by [38] and [39], when the hot-deck procedure is used to generate MI datasets and the same donor pool is employed for all *K* datasets, the method fails to adequately propagate the uncertainty from the predictive distribution. As a result, the variance tends to be underestimated, even when the number of imputations approaches infinity. This underestimation becomes particularly pronounced when a large amount of information is being imputed.

While we are aware of this limitation, we believe that a procedure based on MI method still provides a simple and accessible way to account for matching uncertainty when estimating target parameters. Methods such as the Approximate Bayesian Bootstrap have been proposed to overcome the limitations of the MI method; however, their use by practitioners and in the context of complex sample weights remains largely unexplored.

## Conclusions

In this paper, we have presented ShinyDataMatcher, an interactive R Shiny application designed to support practitioners in implementing statistical matching procedures at both the macro and micro level under the Conditional Independence Assumption (CIA). The app guides the user through the entire workflow of a matching exercise: from data import and initial exploration, through the selection and harmonisation of common variables, to the choice of matching variables, the implementation of a wide range of parametric and non-parametric macro and micro methods, and the evaluation and export of the matched dataset. By integrating these steps into a user-friendly interface and removing the need to write code, ShinyDataMatcher lowers the entry barrier to statistical matching techniques and facilitates their use in applied work.

Although the current version of the application does not yet generate reproducible code, we recognize the importance of transparency and reproducibility in data processing workflows. Code generation is therefore an active component of our ongoing development. In its present form, however, the tool already offers a valuable environment for exploring, prototyping, and testing statistical matching strategies before formal implementation.

The empirical application based on the Italian Household Budget Survey (HBS) and the Survey on Household Income and Wealth (SHIW) illustrates how the proposed tool can be used in realistic settings. We show how the application can be used to harmonise variables from two complex household surveys, select matching variables that are relevant for both income and expenditure, and implement a micro-matching procedure based on random distance hot deck. The resulting matched data were then used to construct a module of the Social Accounting Matrix (SAM) of households by geographical area, with income coming from SHIW and expenditures imputed from HBS, and to quantify the contribution of matching uncertainty through repeated random imputations.

The analysis of the SAM highlights that, for the application considered, matching uncertainty can represent a substantial component of the overall variability of the estimates. Accounting for this additional source of variability leads to wider margins of uncertainty than those obtained when treating the matched dataset as if it were fully observed. Although the approach adopted here is relatively simple, relying on repeated random hot-deck imputations, it provides an accessible way to reflect matching uncertainty in downstream analyses based on fused data.

The current version of ShinyDataMatcher focuses on the case of univariate *Y* and *Z* and on methods that rely on the CIA. Several extensions are possible and left for future work. First, the application could be extended to handle multiple variables of interest in each file and to incorporate methods that explicitly exploit auxiliary information to relax the CIA. Second, additional diagnostic tools and graphical summaries could be incorporated to help users assess the plausibility of modelling assumptions and to compare alternative matching strategies. Finally, from a computational perspective, further developments may include more efficient algorithms for large-scale applications and tighter integration with the workflows of National Statistical Institutes and other producers of official statistics.

Overall, ShinyDataMatcher contributes to bridging the gap between methodological developments in statistical matching and their practical implementation, offering a flexible and transparent environment for integrating survey data and for propagating matching uncertainty to policy-relevant indicators.

## Supporting information

S1 Appendix Technical details on the statistical matching methods. (PDF)

S1 Table Info for matching dataset A. (PDF)

S2 Table Info for matching dataset B. (PDF)

S3 Table Harmonized variables description (Dataset A). (PDF)

S4 Table Harmonized variables description (Dataset B). (PDF)

S1 Fig Some details about the SHIW dataset. In this example, the user is working within the Basic Info tab and is visualizing descriptive statistics for the variable *Y*.
(TIF)

S2 Fig Some details about the HBS dataset. In this example, the user is working within the Variables tab and is visualizing variables name and type.
(TIF)

S3 Fig Selection of relevant information from dataset A (SHIW). In this example, the user selects variables by clicking on the rows of the left-hand table, which lists all available variables, and visualizes the selected information in the right-hand table.
(TIF)

S4 Fig Selection of relevant information from dataset B (HBS). In this example, the user selects variables by clicking on the rows of the left-hand table, which lists all available variables, and visualizes the selected information in the right-hand table.
(TIF)

S5 Fig Recoding of the variable defining geographic areas in dataset A (SHIW). Categories of the original variable are North West, North Est, Central, South and Islands. The user is recoding these categories as NW, NE, C, S, and I, respectively. A view of the original and recoded values is shown in the right-hand table. Note that the variable is renamed as Area in the upper left text input New variable name.
(TIF)

S6 Fig Recoding of the variable defining geographic areas in dataset B (HBS). Categories of the original variable are Nord-ovest, Nord-est, Centro, Sud, and Isole. The user is recoding these categories as NW, NE, C, S, and I, respectively. A view of the original and recoded values is shown in the right-hand table. Note that the variable is renamed as Area in the upper-left text input New variable name. The variable Area is now harmonized.
(TIF)

S7 Fig Recoding of the number of household components variable (NCOMP in Dataset A (SHIW)). The user is grouping the values of the number of household components into the intervals 1, 2, 3, 4, and ≥5. To this aim, the number of categories is set to 5, and the corresponding category labels are entered. As before, the output of the transformation is displayed in the right-hand table.
(TIF)

S8 Fig Recoding of the number of household components variable (c_ncmp_fatto in dataset B (HBS)). The user is making the same transformation as in Figure S7, and is assigning to the transformed variable the same name NCOMP.
(TIF)

S9 Fig Counting the number of employed members of an household (from variables B01_1_Fact to B01_9_Fact in SHIW). The user counts the number of times the category Yes appears for each household and assigns the resulting variable the name Job. Note that the variable can be saved either as a factor or as a numeric variable for subsequent analyses.
(TIF)

S10 Fig Counting the number of employed members of an household (from variables cond_1_Fact to cond_9_Fact in HBS). The user counts the number of times the category Occupato/a appears for each household and assigns the resulting variable the name Job. Note that the variable can be saved either as a factor or as a numeric variable for subsequent analyses.
(TIF)

S11 Fig Users can check the harmonization of the variables. On the left side of the tab, it is possible to see which variables are present in both the donor and the recipient (for example, Area), as well as variables that are present only in one of the two datasets (for example, Y, which is only in the recipient, and Z, which is only in the donor). On the right side, it is possible to verify whether the harmonization of the common variables has been performed correctly, as shown for the variable NCOMP.
(TIF)

S12 Fig Users can see which variables have a significant effect through the output of a regression model on the *Y* variable in dataset A (SHIW), in order to identify variables to be used as matching and/or stratification variables. (TIF)

S13 Fig Users can see which variables have a significant effect through the output of a regression model on the *Z* variable in dataset B (HBS), in order to identify variables to be used as matching and/or stratification variables. (TIF)

S14 Fig On the left side of the tab, users can select the options to be used for micro-matching. In this case, the variable to be imputed is Z, and the selected matching variables are Degree, Diploma, Job, and Ret. Users can also specify whether donation classes should be used and select the corresponding variables, in this case Area and NCOMP. The distance measure can be specified (e.g., Manhattan), together with the number of matching variables to be generated. On the right side, dataset A with the generated imputed variable is reported.
(TIF)

S15 Fig Users can store the generated matching variables, which will be linked to the original SHIW dataset. Once the imputed variables have been stored, users can download the original SHIW dataset, which also includes the newly imputed variables.
(TIF)
