# Supplementary material for: ShinyDataMatcher: A user-friendly application for integrating survey data
Source: PLoS One. 2026 Jul 14;21(7):e0353530. doi: 10.1371/journal.pone.0353530 (PMC13367710; doi:10.1371/journal.pone.0353530)
Supplement: S1 Appendix — (PDF) [file pone.0353530.s001.pdf]

Technical Details on Statistical Matching Methods

S1 Parametric Methods

S1.1 Macro Methods

For the macro approach, the model is identifiable under the CIA, and maximum likelihood (ML) estimates of the parameters can be obtained by maximizing the following likelihood [1]:

$$\begin{aligned}\mathscr{L}(\boldsymbol{\theta}|A\cup B) &= \prod_{a=1}^{n_A} f_{\mathbf{X}Y}(\mathbf{x}_a, y_a;\boldsymbol{\theta}) \prod_{b=1}^{n_B} f_{\mathbf{X}Z}(\mathbf{x}_b, z_b;\boldsymbol{\theta}) \\ &= \prod_{a=1}^{n_A} f_{Y|\mathbf{X}}(y_a|\mathbf{x}_a;\boldsymbol{\theta}_{Y|\mathbf{X}}) f_{\mathbf{X}}(\mathbf{x}_a;\boldsymbol{\theta}_{\mathbf{X}}) \times \\ &\quad \times \prod_{b=1}^{n_B} f_{Z|\mathbf{X}}(z_b|\mathbf{x}_b;\boldsymbol{\theta}_{Z|\mathbf{X}}) f_{\mathbf{X}}(\mathbf{x}_b;\boldsymbol{\theta}_{\mathbf{X}}),\end{aligned} \tag{S1}$$

where the marginal and joint densities of $\mathbf{X}, Y, Z$ depend on the nature of the variables.

When dealing with continuous variables, multivariate normality is often assumed. In this case, the distribution is parameterised by the mean vector $\boldsymbol{\mu}$ and covariance matrix $\boldsymbol{\Sigma}$:

$$\boldsymbol{\theta} = (\boldsymbol{\mu}, \boldsymbol{\Sigma}) = \left[\begin{pmatrix}\boldsymbol{\mu}_{\mathbf{X}} \\ \mu_Y \\ \mu_Z\end{pmatrix}, \begin{pmatrix}\boldsymbol{\Sigma}_{\mathbf{X}} & \boldsymbol{\Sigma}_{\mathbf{X}Y} & \boldsymbol{\Sigma}_{\mathbf{X}Z} \\ \boldsymbol{\Sigma}_{Y\mathbf{X}} & \sigma_Y^2 & \sigma_{YZ} \\ \boldsymbol{\Sigma}_{Z\mathbf{X}} & \sigma_{ZY} & \sigma_Z^2\end{pmatrix}\right]. \tag{S2}$$

Let $\mathbf{w} = \left(\mathbf{x}^\top, y, z\right)^\top$. The joint density of $(\mathbf{X}, Y, Z)$ is:

$$f(\mathbf{w}|\boldsymbol{\mu}, \boldsymbol{\Sigma}) = (2\pi)^{-\frac{L+2}{2}} |\boldsymbol{\Sigma}|^{-\frac{1}{2}} \exp\left\{-\frac{1}{2}(\mathbf{w}-\boldsymbol{\mu})^\top \boldsymbol{\Sigma}^{-1}(\mathbf{w}-\boldsymbol{\mu})\right\}, \tag{S3}$$

with $\mathbf{w} \in \mathbb{R}^{L+2}$. Note that, under the CIA, $\sigma_{YZ} = \boldsymbol{\Sigma}_{Y\mathbf{X}}\boldsymbol{\Sigma}_{\mathbf{X}}^{-1}\boldsymbol{\Sigma}_{\mathbf{X}Z}$.

By the properties of the multivariate normal distribution [2], the marginal distribution of $\mathbf{X}$ is normal with parameters $\boldsymbol{\theta}_{\mathbf{X}} = (\boldsymbol{\mu}_{\mathbf{X}}, \boldsymbol{\Sigma}_{\mathbf{X}})$ that can be estimated on the pooled dataset $A\cup B$ with the ML estimators:

$$\hat{\boldsymbol{\mu}}_{\mathbf{X}} = \bar{\mathbf{x}}_{A\cup B} = \frac{1}{n_A+n_B}\left(\sum_{a=1}^{n_A}\mathbf{x}_a + \sum_{b=1}^{n_B}\mathbf{x}_b\right), \tag{S4}$$

$$\hat{\boldsymbol{\Sigma}}_{\mathbf{X}} = \frac{1}{n_A+n_B}\left[\sum_{a=1}^{n_A}(\mathbf{x}_a-\hat{\boldsymbol{\mu}}_{\mathbf{X}})(\mathbf{x}_a-\hat{\boldsymbol{\mu}}_{\mathbf{X}})^\top + \sum_{b=1}^{n_B}(\mathbf{x}_b-\hat{\boldsymbol{\mu}}_{\mathbf{X}})(\mathbf{x}_b-\hat{\boldsymbol{\mu}}_{\mathbf{X}})^\top\right]. \tag{S5}$$

The conditional distribution of $Y|\mathbf{X}$ is normal and depends on the parameters $\boldsymbol{\theta}_{Y|\mathbf{X}} = (\boldsymbol{\mu}_{Y|\mathbf{X}}, \boldsymbol{\Sigma}_{Y|\mathbf{X}})$. It can be equivalently characterized through the regression model:

$$Y = \alpha_{Y|\mathbf{X}} + \mathbf{X}^\top \widetilde{\boldsymbol{\beta}}_{Y|\mathbf{X}} + \varepsilon_{Y|\mathbf{X}}, \tag{S6}$$

where $\varepsilon_{Y|\mathbf{X}} \sim \mathcal{N}\left(0, \sigma^2_{Y|\mathbf{X}}\right)$, the conditional variance of Y given $\mathbf{X}$ is denoted as $\sigma^2_{Y|\mathbf{X}}$, while $\alpha_{Y|\mathbf{X}}$ and $\widetilde{\boldsymbol{\beta}}_{Y|\mathbf{X}} \in \mathbb{R}^L$ denote the intercept term and the regression coefficients of Y on $\mathbf{X}$, respectively.
Let $\widetilde{\mathbf{X}}^A = \left[\mathbf{1}_{n_A}\ \mathbf{X}^A\right]$, where $\mathbf{1}_{n_A}$ is the n_A-dimensional vector of ones. The ML estimates of the parameter vector $\boldsymbol{\beta}_{Y|\mathbf{X}} = \left(\alpha_{Y|\mathbf{X}}, \widetilde{\boldsymbol{\beta}}^\top_{Y|\mathbf{X}}\right)^\top$ coincide with the Ordinary Least Squares (OLS) estimator:

$$\hat{\boldsymbol{\beta}}^A_{Y|\mathbf{X}} = \left(\widetilde{\mathbf{X}}^{A\top}\widetilde{\mathbf{X}}^A\right)^{-1}\widetilde{\mathbf{X}}^{A\top}\mathbf{y}^A. \tag{S7}$$

The conditional variance $\sigma^2_{Y|\mathbf{X}}$ can be estimated from the residual sum of squares as:

$$\hat{\sigma}^2_{Y|\mathbf{X}} = \frac{1}{n_A}\left(\mathbf{y}^A - \hat{\mathbf{y}}^A\right)^\top\left(\mathbf{y}^A - \hat{\mathbf{y}}^A\right), \tag{S8}$$

where $\hat{\mathbf{y}}^A = \widetilde{\mathbf{X}}^A\hat{\boldsymbol{\beta}}^A_{Y|\mathbf{X}}$. Under the CIA, the parameters of the joint distribution (S2) can be estimated as follows:

$$\hat{\mu}_Y = \hat{\alpha}_{Y|\mathbf{X}} + \hat{\boldsymbol{\beta}}_{Y|\mathbf{X}}\hat{\boldsymbol{\mu}}_{\mathbf{X}}, \tag{S9}$$

$$\hat{\sigma}^2_Y = \hat{\sigma}^2_{Y|\mathbf{X}} + \hat{\boldsymbol{\Sigma}}_{Y\mathbf{X}}\hat{\boldsymbol{\Sigma}}^{-1}_{\mathbf{X}}\hat{\boldsymbol{\Sigma}}_{\mathbf{X}Y}, \tag{S10}$$

$$\hat{\boldsymbol{\Sigma}}_{Y\mathbf{X}} = \hat{\boldsymbol{\beta}}_{Y\mathbf{X}}\hat{\boldsymbol{\Sigma}}_{\mathbf{X}}. \tag{S11}$$

Analogous considerations hold for $Z|\mathbf{X}$, and therefore the parameters $\boldsymbol{\mu}_Z$, $\boldsymbol{\Sigma}_Z$ and $\boldsymbol{\Sigma}_{Z\mathbf{X}}$ can be estimated with the same procedure. Alternative estimation methods to ML have been explored in the literature. For example, [3] suggests using least squares estimators for the regression parameters, which produce results close to those obtained from ML, particularly with large sample sizes. In another approach, [4] estimates $\boldsymbol{\theta}$ by utilizing its observed sample counterpart.

When dealing with categorical variables, for simplicity of exposition we assume that X is a scalar variable. The results can be extended to the multivariate case, with details available in [5]. If (X, Y, Z) has a categorical distribution with categories $H \times J \times K$, the parameter vector θ_{hjk}, for $h = 1, \ldots, H$, $j = 1, \ldots, J$ and $k = 1, \ldots, K$, is:

$$\theta_{hjk} = \Pr\left\{X = h, Y = j, Z = k\right\}, \quad h = 1, \ldots, H;\ j = 1, \ldots, J;\ k = 1, \ldots, K,$$
$$\theta_{hjk} \geq 0 \quad \forall h, j, k, \qquad \sum_{h=1}^{H}\sum_{j=1}^{J}\sum_{k=1}^{K}\theta_{hjk} = 1.$$

Under the CIA, $\boldsymbol{\theta} = (\boldsymbol{\theta}_X, \boldsymbol{\theta}_{Y|X}, \boldsymbol{\theta}_{Z|X})$, where $\boldsymbol{\theta}_X = \{\theta_{h..}\}$, $\boldsymbol{\theta}_{Y|X} = \{\theta_{j|h} = \theta_{hj.}/\theta_{h..}\}$, $\boldsymbol{\theta}_{Z|X} = \{\theta_{k|h} = \theta_{h.k}/\theta_{h..}\}$, where a dot in the subscript indicates that the variable has been marginalized. The parameter of the joint distribution of (X, Y, Z) can be expressed as a product of marginal and conditional probabilities as

$$\theta_{hjk} = \theta_{h..}\theta_{j|h}\theta_{k|h} = \frac{\theta_{hj.}\theta_{h.k}}{\theta_{h..}}, \tag{S12}$$

and the parameters of the joint distribution of Y and Z can be obtained by marginalizing with respect to

X as

$$\theta_{.jk} = \sum_{h=1}^{H} \theta_{j|h}\theta_{k|h}\theta_{h..} \qquad j = 1,\ldots,J, \quad k = 1,\ldots,K. \tag{S13}$$

ML estimates of $\boldsymbol{\theta}$, obtained by maximizing the likelihood function in (S1), have the following form:

$$\hat{\theta}_{h..} = \frac{n^A_{h..} + n^B_{h..}}{n_A + n_B}, \qquad h = 1,\ldots,H, \tag{S14}$$

$$\hat{\theta}_{j|h} = \frac{n^A_{hj.}}{n^A_{h..}}, \qquad h = 1,\ldots,H;\ j = 1,\ldots,J, \tag{S15}$$

$$\hat{\theta}_{k|h} = \frac{n^B_{h.k}}{n^B_{h..}}, \qquad h = 1,\ldots,H;\ k = 1,\ldots,K, \tag{S16}$$

where $n^A_{h..}$ and $n^B_{h..}$ are the observed marginal frequencies of X that can be obtained from datasets A and B, respectively; $n^A_{hj.}$ represents the observed frequencies of the categories (h, j) in the marginal distribution of X and Y in dataset A, and $n^B_{h.k}$ represents the observed frequencies of the categories (h, k) in the marginal distribution of X and Z in dataset B.

The ML estimates of the parameters of the joint distribution of (X, Y, Z) and of the distribution of (Y, Z) can be obtained by substituting $\hat{\theta}_{h..}$, $\hat{\theta}_{j|h}$, and $\hat{\theta}_{k|h}$ into (S12) and (S13).

S1.2 Micro Methods

A complete synthetic dataset can be obtained by estimating the regression model of Y on $\mathbf{X}$ from dataset A and the regression model of Z on $\mathbf{X}$ from dataset B. We consider the imputation of the missing variable Z in dataset A; however, the same theory can also be applied to the imputation of the missing variables Y in dataset B. The imputation can then be performed using two parametric methods: conditional mean matching and stochastic regression imputation.

Conditional mean matching consists of substituting each missing value with the expected value of the missing variables, given the observed variables. When the distribution of the variables is multivariate normal, maximum likelihood (ML) estimates of $\boldsymbol{\theta}_{Z|\mathbf{X}}$ can be obtained as described in the Macro Methods Section, allowing us to compute the imputed variables $\tilde{\boldsymbol{z}}^A$. Due to the duality between the multivariate normal distribution and regression models, this is also a classic regression imputation method [6], where

$$\tilde{\boldsymbol{z}}^A = \widetilde{\mathbf{X}}^A \hat{\boldsymbol{\beta}}^B_{Z|\boldsymbol{X}}. \tag{S17}$$

A drawback of this method is that the predicted values do not account for the variability of the imputed variable around its conditional mean. To address this limitation, random values can be drawn from $f_{\boldsymbol{Z}|\boldsymbol{X}}(\boldsymbol{z}|\boldsymbol{x}_a; \hat{\boldsymbol{\theta}}_{\boldsymbol{Z}|\boldsymbol{X}})$, for every $a = 1,\ldots,n_A$. When the distribution of $(\boldsymbol{X}, \boldsymbol{Y}, \boldsymbol{Z})$ is multivariate normal, this process is known as stochastic regression imputation [6], where the imputed variable is obtained as:

$$\tilde{\boldsymbol{z}}^A = \widetilde{\mathbf{X}}^A \hat{\boldsymbol{\beta}}^B_{Z|\boldsymbol{X}} + \mathbf{e}_{Z|\mathbf{X}}, \tag{S18}$$

where $\mathbf{e}_{Z|\mathbf{X}}$ is randomly generated from a multivariate normal with mean vector $\mathbf{0}$ and covariance matrix $\hat{\sigma}^2_{Z|\mathbf{X}}\mathbf{I}_{n_A}$.

S2 Non-parametric methods

Non-parametric methods at the micro level are referred to as hot-deck methods [7]. In general, for all micro-level methods, dataset A serves as the recipient file, while dataset B is the donor file. Missing values for the variable $\boldsymbol{Z}$ in the recipient dataset are filled with observed values from the donor dataset, rather than with predicted values as in parametric micro methods. Typically, $n_B \geq n_A$. The final matched synthetic dataset consists of n_A units and includes the values $\boldsymbol{x}$, $\boldsymbol{y}$, and $\tilde{\boldsymbol{z}}$, where $\tilde{\boldsymbol{z}}$ has been imputed. The main techniques applied under the CIA are listed below.

- **Random hot deck method.** This method involves randomly selecting a donor record from the donor file for each record in the recipient file. The number of possible donor assignments is $n_B^{n_A}$.

  Sometimes, this selection is done within specific subsets of units within the donor files using one or a few categorical variables shared between both files, such as demographic variables, forming what are known as donation classes [7]. Given a categorical variable X with C categories (i.e., $c = 1,\dots,C$), let $n_A(c)$ denote the number of units in set A that belong to category c and $n_B(c)$ denote the number of units in set B that belong to category c. The number of possible donor assignments is then given by $n_{B(c)}^{n_{A(c)}}$.

  The method can be constrained, meaning each donor is used only once, or unconstrained, allowing each donor to be selected multiple times.

- **Rank hot deck method.** In the rank hot deck method, an ordinal matching variable X is used to select donors for assignment to records in dataset A [8]. Units in both datasets are ranked independently based on their values of X. When $n_B = kn_A$ (with k an integer), matching is achieved by pairing records with the same rank. If the sample sizes of A and B are unequal, matching is done by aligning records according to the empirical cumulative distribution functions of X in both the recipient and donor files:

$$\hat{F}_X^A(x) = \frac{1}{n_A}\sum_{a=1}^{n_A} I(x_a \leq x), \quad x \in \mathcal{X}, \qquad \hat{F}_X^B(x) = \frac{1}{n_B}\sum_{b=1}^{n_B} I(x_b \leq x), \quad x \in \mathcal{X}. \tag{S19}$$

  Subsequently, each $a = 1,\dots,n_A$ is linked to the corresponding record b^* in B such that [5]

$$\left|\hat{F}_X^A(x_a^A) - \hat{F}_X^B(x_{b^*}^B)\right| = \min_{1\leq b\leq n_B} \left|\hat{F}_X^A(x_a^A) - \hat{F}_X^B(x_b^B)\right|. \tag{S20}$$

- **Distance hot deck method.** In the unconstrained distance method, with a single continuous matching variable X, the donor for the a-th record in the recipient file A is selected so that [9]

$$d_{ab^*} = |x_a^A - x_{b^*}^B| = \min_{1\leq b\leq n_B} |x_a^A - x_b^B|. \tag{S21}$$

  The procedure is unconstrained, as each record in the donor file B can be used as a donor multiple times.

  In the constrained distance methods, each record in B can be chosen as a donor only once, which requires $n_A \leq n_B$. The constrained distance hot deck method is computationally expensive, particularly for large datasets. However, it has the advantage of preserving the marginal distribution of the imputed variable Z in the recipient dataset, especially when n_A and n_B are equal.

Distance-based hot deck techniques are flexible, accommodating various metrics. For numerical variables $\boldsymbol{X}$, the distance between a unit b in dataset B and a unit a in dataset A can be computed using classical distance measures, such as the Euclidean, Manhattan, and Mahalanobis distances [10]. For categorical variables, the Jaccard index is commonly used [11]. In the presence of mixed numerical and categorical variables, the Gower distance provides a flexible and widely adopted solution [12].

References

[1] Rubin DB. Characterizing the estimation of parameters in incomplete-data problems. Journal of the American Statistical Association. 1974;69(346):467-74.

[2] Anderson TW, Anderson TW, Anderson TW, Anderson TW. An introduction to multivariate statistical analysis. vol. 2. Wiley New York; 1958.

[3] Rässler S. Statistical matching: A frequentist theory, practical applications, and alternative Bayesian approaches. vol. 168. Springer Science & Business Media; 2012.

[4] Moriarity C, Scheuren F. Statistical matching: a paradigm for assessing the uncertainty in the procedure. Journal of Official Statistics. 2001;17(3):407.

[5] D'Orazio M, Di Zio M, Scanu M. Statistical matching: Theory and practice. John Wiley & Sons; 2006.

[6] Little RJ, Rubin DB. Statistical analysis with missing data. vol. 793. John Wiley & Sons; 2019.

[7] Singh A, Mantel H, Kinack M, Rowe G. Statistical matching: use of auxiliary information as an alternative to the conditional independence assumption. Survey Methodology. 1993;19(1):59-79.

[8] Singh A, Mantel H, Kinack M, Rowe G. On methods of statistical matching with and without auxiliary information. Methodology Branch, Statistics Canada (Technical Report SSMD-90-016E) Available at: http://publications gc ca/collections/collection_2017/statcan/11-613/CS11-613-90-16-eng pdf (accessed April 2015). 1990.

[9] Okner B. Constructing a new data base from existing microdata sets: the 1966 merge file. In: Annals of Economic and Social Measurement, Volume 1, Number 3. NBER; 1972. p. 325-62.

[10] Mahalanobis PC. On the Generalised Distance in Statistics. Proceedings of the National Institute of Sciences of India. 1936;2(1):49-55.

[11] Jaccard P. Étude comparative de la distribution florale dans une portion des Alpes et des Jura. Bulletin de la Société Vaudoise des Sciences Naturelles. 1901;37:547-79.

[12] Gower JC. A General Coefficient of Similarity and Some of Its Properties. Biometrics. 1971;27(4):857-71.
