# Supplementary material for: ShinyDataMatcher: A user-friendly application for integrating survey data
Source: PLoS One. 2026 Jul 14;21(7):e0353530. doi: 10.1371/journal.pone.0353530 (PMC13367710; doi:10.1371/journal.pone.0353530)
Supplement: S1 Table — (PDF) [file pone.0353530.s002.pdf]

Variable	Description	Categories/Range of values
Y	Net disposable income	$\mathbb{R}_0^+$
STUDIO_1_Fact-STUDIO_9_Fact	Educational qualification of each member	1="none", 2="elementary school certificate", 3="lower middle school certificate", 4="professional diploma (3 years)", 5="high school diploma", 6="university diploma/bachelor's degree", 7="master's degree", 8="postgraduate specialization"
B01_1_Fact-B01_9_Fact	Employed	1="Yes",2="No"
APQUAL2_1_Fact-APQUAL2_9_Fact	Professional position of each member	1="Worker or similar position (including salaried employees, apprentices, home workers, shop assistants)", 2="Clerk", 3="Teacher of any type of school (including temporary or contract teachers)", 4="Executive/managerial clerk", 5="Manager, senior official, headmaster, principal, university professor, magistrate", 6="Freelancer", 7="Individual entrepreneur", 8="Self-employed worker (e.g., craftsman)", 9="Owner, partner, or manager of a company or cooperative", 10="Family assistant (not employed) in the business of a cohabiting family member", 11="Looking for first job", 12="Unemployed", 13="Homemaker", 14="Wealthy individual", 15="Retired (work-related pension)", 16="Retired (non-work-related: disability/survivor/social pension)", 17="Student (from primary school onward)", 18="Preschool child", 19="Volunteer", 20="Non-standard worker (freelancer, occasional collaborator, accessory worker, family assistant not employed in the business of a non-cohabiting family member or friend, etc.)", 21="Other non-employed"
AREA5_1_Fact	Geographic area of residence	1="North West", 2="North East", 3="Central", 4="South", 5="Islands"
SUPAB	What is the surface area (in square meters) of this dwelling/apartment?	$\mathbb{R}^+$
NCOMP	Number of people living in this household, 0 years or older, as of 31-12-2020	N^+
