# Supplementary material for: ShinyDataMatcher: A user-friendly application for integrating survey data
Source: PLoS One. 2026 Jul 14;21(7):e0353530. doi: 10.1371/journal.pone.0353530 (PMC13367710; doi:10.1371/journal.pone.0353530)
Supplement: S2 Table — (PDF) [file pone.0353530.s003.pdf]

Variable	Description	Categories/Range
sp_tot_str_aggr_1	Total Household Expenditure	$\mathbb{R}_0^+$
c_titstu_1_Fact-c_titstu_12_Fact	Education level of each component	1="No qualification", 2= "Elementary school", 3="Middle school", 4="High school", 5="University degree and postgraduate"
cond_1_Fact-cond_12_Fact	Condition of each component	1="Employed", 2="Seeking employment", 3="Housework/student/other", 4="Retired"
rip_Fact	Geographical region (5 level)	1="North-west", 2="North-east", 3="Centre", 4="South", 5="Islands"
c_superf	Area of the dwelling	$\mathbb{R}^+$
c_ncmp_fatto	Total number of members in the cohabiting family	N^+
