# Supplementary material for: ShinyDataMatcher: A user-friendly application for integrating survey data
Source: PLoS One. 2026 Jul 14;21(7):e0353530. doi: 10.1371/journal.pone.0353530 (PMC13367710; doi:10.1371/journal.pone.0353530)
Supplement: S3 Table — (PDF) [file pone.0353530.s004.pdf]

Variable(s) I_A	Transformation Selected	New Name	Description	Categories/Range
Y	Add at it is	Y	Net disposable income	$\mathbb{R}_0^+$
AREA5_1_Fact	Recode the level of a factor	Area	Geographic area of residence	"NE", "NW", "C", "S", "I"
STUDIO_1_-Fact-STUDIO_-9_Fact	Count the number of occurrences	No_tit	No. of members with no school qualifications	N
STUDIO_1_-Fact-STUDIO_-9_Fact	Count the number of occurrences	Comp	No. of members with up to 8 years' schooling	N
STUDIO_1_-Fact-STUDIO_-9_Fact	Count the number of occurrences	Diploma	No. of members with 9–13 years' schooling	N
STUDIO_1_-Fact-STUDIO_-9_Fact	Count the number of occurrences	Degree	No. of members with a university degree	N
B01_1_-Fact-B01_9_-Fact	Count the number of occurrences	Job	No. of members with a job	N
APQUAL2_1_-Fact-APQUAL2_-9_Fact	Count the number of occurrences	Ret	No. of members retired	N
SUPAB	Rename a variable	Housesup	Total surface area of house (m^2)	R^+
NCOMP	Quantitative to categorical	NCOMP	Number of members of the household	"1", "2", "3", "4", ">=5"
