# Supplementary material for: ShinyDataMatcher: A user-friendly application for integrating survey data
Source: PLoS One. 2026 Jul 14;21(7):e0353530. doi: 10.1371/journal.pone.0353530 (PMC13367710; doi:10.1371/journal.pone.0353530)
Supplement: S4 Table — (PDF) [file pone.0353530.s005.pdf]

Variable(s) I_B	Transformation Selected	New Name	Description	Categories/Range
`sp_tot_str_-aggr_1`	Rename Variable	`Z`	Total Household Expenditure	R^+
`rip_fact`	Recode the level of a factor	`Area`	Geographic area of residence	"NE", "NW", "C", "S", "I"
`c_titstu_-1_Fact-c_-titstu_12_Fact`	Count the number of occurrences	`No_tit`	No. of members with no school qualifications	N
`c_titstu_-1_Fact-c_-titstu_12_Fact`	Count the number of occurrences	`Comp`	No. of members with up to 8 years' schooling	N
`c_titstu_-1_Fact-c_-titstu_12_Fact`	Count the number of occurrences	`Diploma`	No. of members with 9–13 years' schooling	N
`c_titstu_-1_Fact-c_-titstu_12_Fact`	Count the number of occurrences	`Degree`	No. of members with a university degree	N
`cond_1_-Fact-cond_-12_Fact`	Count the number of occurrences	`Job`	No. of members with a job	N
`cond_1_-Fact-cond_-12_Fact`	Count the number of occurrences	`Ret`	No. of members retired	N
`c_Superf`	Rename a variable	`Housesup`	Total surface area of house (m^2)	R^+
`c_Ncmp_fatto`	Quantitative to categorical	`NCOMP`	Number of members of the household	"1", "2", "3", "4", ">=5"
