# Supplementary material for: ShinyDataMatcher: A user-friendly application for integrating survey data
Source: PLoS One. 2026 Jul 14;21(7):e0353530. doi: 10.1371/journal.pone.0353530 (PMC13367710; doi:10.1371/journal.pone.0353530)
Supplement: Attachment — Submitted filename: review.pdf [file pone.0353530.s021.pdf]

In general, I like the paper and the app. I think it is relevant and intuitive for people who are interested in statistical matching. I mainly have some suggestions for the construction of the paper. I hope the paper can therefore be more attractive to our readers.

Major:

1. I think it would be better to try to make the theory of statistical matching more concise, and mainly focus on the app itself. After all, the app is the star of this paper and we would not want to exhaust our readers before they really get to the important part. One option may be to leave the technical details of statistical matching to the supplement and only keep the general idea and assumptions of statistical matching in the main text.

2. The workflow has been introduced many times in the section of The workflow of Statistical Matching, ShinyDataMatcher: Overview, and Application. I believe these can be combined or trimmed in a way to overall shorten the paper.

3. As a potential user, I will be interested in how you make the decisions in the application. For example, why you choose these variables for matching, why you harmonize in this way, and why you choose Random Distance Hot Deck.

4. The formula for the variance of multiple imputation is incorrect. It will be better to cite the variance calculation from Rubin (1987) instead of Rubin (1978). Rubin (1996) may also be a nice reference.

Minor:

1. I tried to download the shiny app. The code **remotes::install github(fedele-greco/ShinyStatMatcher)** does not work for me, but **remotes::install github("fedele-greco/ShinyStatMatcher")** does.

2. In the Application, lots of figures and tables are left in the supplement. I personally find it quite hard to read. Perhaps we can only keep some of the tabs and put them in the main text? I do understand that you may want to keep them as a tutorial. But the app itself is already quite self-explanatory in my opinion.

3. **PARENT_- 1_Fact** to **PARENT_- 9_Fact** in table S1 cannot be found in SHIW, and **ID, c_relaz_1_-Fact** to **c_relaz_12_-Fact** in table S2 cannot be found in HBS. Therefore, I did not continue trying out the application. I will be happy to do so if there is another round of review.

Reference

Rubin, D. B. (1987). Multiple imputation for nonresponse in surveys. New York, NY.

Rubin, D. B. (1996). Multiple imputation after 18+ years. Journal of the American statistical Association, 91(434), 473-489.
