# Supplementary material for: ShinyDataMatcher: A user-friendly application for integrating survey data
Source: PLoS One. 2026 Jul 14;21(7):e0353530. doi: 10.1371/journal.pone.0353530 (PMC13367710; doi:10.1371/journal.pone.0353530)
Supplement: Attachment — Submitted filename: response reviewers.pdf [file pone.0353530.s024.pdf]

April 17, 2026

Object: PONE-D-26-04293 revised paper submission

Dear Reviewers,

we thank you for carefully reading our paper and highlighting critical points. We hope that this new version improves our work in the direction you indicated. Changes to the previous version have been highlighted in bold in order to facilitate your reading of the revised paper. Please find a point-by-point answer to your comments in what follows (our answers are interlined to your suggestions).

Reply to the Reviewer # 1

Thank you very much for your valuable comments and suggestions. We have carefully considered them and made revisions to address your concerns in this paper version.

1. *I think it would be better to try to make the theory of statistical matching more concise, and mainly focus on the app itself. After all, the app is the star of this paper and we would not want to exhaust our readers before they really get to the important part. One option may be to leave the technical details of statistical matching to the supplement and only keep the general idea and assumptions of statistical matching in the main text.*
   Following your suggestion, we have substantially streamlined the *Statistical Framework and Methods* section. The main text now provides only a general overview of statistical matching, while the technical details have been moved to Appendix S1. In particular, the description of parametric macro methods is now presented in Section S1.1, parametric micro methods in Section S1.2, and non-parametric micro methods in Section S2 of the appendix.

2. *The workflow has been introduced many times in the section of The workflow of Statistical Matching, ShinyDataMatcher: Overview, and Application. I believe these can be combined or trimmed in a way to overall shorten the paper*
   Following your suggestion, we have created a single section that summarizes the information previously presented in *The Workflow of Statistical Matching* and *ShinyDataMatcher: Overview*. This section is now titled *Statistical matching through ShinyDataMatcher*. However, we have chosen to keep the real data application in a separate section due to its importance, as it can also serve as a tutorial to use the app for users.

3. *As a potential user, I will be interested in how you make the decisions in the application. For example, why you choose these variables for matching, why you harmonize in this way, and why you choose Random Distance Hot Deck*
   Thank you for this important comment. We have clarified the rationale behind the main choices made in the application.

   First, the empirical application is designed to replicate the framework of D'Orazio et al. (2006), using more recent data (year 2020). This has now been explicitly stated at the beginning of the Real Data Application section.

   Second, the selection of variables is guided by standard practice in statistical matching: users should choose variables that capture comparable information across datasets (e.g., demographic and socio-economic characteristics). In our case, we retained a subset of variables from D'Orazio et al. (2006) to simplify the illustration. This has been clarified in the manuscript.

   Third, the harmonization strategy follows the same reference, with additional explanation provided in the revised text. In particular, we clarify why aggregation procedures (e.g., counting occurrences) are used to preserve information at the household level.

Finally, we have clarified the choice of the Random Distance Hot Deck method. This method allows the generation of multiple imputations, which is essential for assessing matching uncertainty. Compared to the standard random hot-deck, it also provides more accurate matches by incorporating a distance metric.

All these clarifications have been added in the relevant sections of the manuscript (section *Real Data application*).

4. *The formula for the variance of multiple imputation is incorrect. It will be better to cite the variance calculation from Rubin (1987) instead of Rubin (1978). Rubin (1996) may also be a nice reference*
In the subsection *Building the SAM matrix*, we have corrected the formula of the variance of the multiple imputation (MI) following Rubin (1996). The variance is now specified as:

$$\hat{V}(\bar{Z}) = W + \left(\frac{K+1}{K}\right) B, \tag{1}$$

where the formula for the between-imputation variance has been corrected as follows:

$$B = \frac{1}{K-1} \sum_{k=1}^{K} \left(\bar{Z}^{(k)} - \bar{Z}\right)^2. \tag{2}$$

The results in Table 4 have been modified accordingly.

Minor:

1. *I tried to download the shiny app. The code remotes::install github(fedele-greco/ShinyStatMatcher) does not work for me, but remotes::install github("fedele-greco/ShinyStatMatcher") does.*
We have corrected the command line to `remotes::install_github("fedele-greco/ShinyStatMatcher")` in line 60 of the paper.

2. *In the Application, lots of figures and tables are left in the supplement. I personally find it quite hard to read. Perhaps we can only keep some of the tabs and put them in the main text? I do understand that you may want to keep them as a tutorial. But the app itself is already quite self-explanatory in my opinion.*
In response to this comment, we have moved all essential information required to perform the real data application into the main text. First, more details on how to actually perform the application, including all the specific features and fields to use, have been added in the section *Real Data Application* Section. Tables 1 and 2 have been inserted in the subsection *Select relevant information for matching* and include all the raw variables to be selected for matching. A more detailed version of these tables has been included as supplementary material (Tables S1 and S2). The harmonization phase, described in detail, has also been included in the main text. Tables S3 and S4 have been retained in the supplementary material as a cross-check. Table S5 has been removed, as all the relevant information is now included in the main text. We decided to keep the screenshots in the supplementary material, as they are not essential for actually performing the application.

3. *PARENT_- 1_Fact to PARENT_- 9_Fact in table S1 cannot be found in SHIW, and ID, c_relaz_1_-Fact to c_relaz_12_-Fact in table S2 cannot be found in HBS. Therefore, I did not continue trying out the application. I will be happy to do so if there is another round of review.*
   In the previous version of the paper, we made an error in the list of raw variables to be selected in SHIW and HBS, by including some that were not actually useful for the application at hand, as those that you cited in your comment. We have now created the correct tables (Tables 1 and 2), which have been inserted in the main text, along with more detailed versions provided in the supplementary material (Tables S1 and S2).

Reply to the Reviewer # 2
Thank you very much for your valuable comments and suggestions. We have carefully considered them and made revisions to address your concerns in this paper version.

Reply to Minor Comments

1. *Page 2/21, line 60: There is a missing quotation mark in the command: remotes::install_github('fedele-greco/ShinyStatMatcher')*
   We have corrected the command line to `remotes::install_github("fedele-greco/ShinyStatMatcher")` in line 60 of the paper.

2. *Page 8/21: It is unclear what is meant by "information." Does this refer only to the raw variables in the dataset that are used to construct the final harmonized variables for matching, to survey design characteristics, or to correlations between variables? Later, _ and _ are denoted as reduced datasets containing candidate variables for matching. This is confusing and should be clarified*
   The term information refers to the raw variables selected from the original datasets that will play a role in the matching procedure, for example as target variables or as variables to be harmonized for matching. The reduced datasets that include these selected raw variables are denoted as $\mathcal{I}_A$ and $\mathcal{I}_B$. We have emphasized this point in the subsection *Select Relevant Information from A and B* of the section *Statistical Matching through ShinyDataMatcher*.

   The other notation we use refers to the harmonized datasets, denoted as $\mathbf{X}_A$ and $\mathbf{X}_B$. These datasets include candidate matching variables that have been harmonized, as well as the target variables. We have clarified this aspect in the subsection *Variables Harmonization* of the section *Statistical Matching through ShinyDataMatcher*.

3. *Page 10/21: "List of Available Surveys." -> Replace the period with a colon for consistency*
   We have replaced the period with a colon.

4. *Page 13/21: I find the terminology "key variable," used to refer to unit IDs in the dataset, somewhat confusing. I suggest renaming it to make explicit that it refers to the unit identifier.*
   We have replaced the terminology "key variable" with "ID variable" in both the paper and the app.

5. *Figure 7: is difficult to read due to too many overlapping arrows and squares* Following your suggestion, we have split Figure 7 into two figures. Figure 7 now includes only the statistical macro matching methods, while Figure 8 presents only the micro methods.

Reply to Major comments on the application section

1. *The selection of relevant "information" remains unclear. Tables S1 and S2 present the list of variables to be selected for matching; however, not all of these variables appear to be available in the dataset. The manuscript should specify explicitly which variables should be selected*
   We thank the reviewer for this observation. In the previous version, the list of variables included some inconsistencies. We have now revised the selection of variables and provided a clear and correct list in Tables 1 and 2 in the main text. These tables explicitly indicate the variables required to replicate the application. Additional details are provided in Tables S1 and S2 in the supplementary material.

2. *Under Data Wrangling -> Select a transformation (one variable): while multiple options can be selected, the tool does not appear to be designed to operate with multiple transformations simultaneously. In addition, when selecting "quantitative to categorical," the number of categories can drop below two, even becoming negative. This should be corrected.*
   Thank you for pointing this out. The tool is not designed to operate with multiple transformations simultaneously. We have modified the app, and it is now possible to select only one transformation at a time. If a single variable is selected in the field `Select a variable (or a group of variables)`, the menu `Select a transformation (one variable)` will appear with the following options: `Add as it is`, `Rename variable`, `Quantitative to categorical`, and `Recode a factor`.
   To visualize the transformation when multiple variables are selected, users need to select more than one variable in the field `Select a variable (or a group of variables) to transform` in one of the tabs of data wrangling. In this case, a menu `Select a transformation (several variables)` will appear, offering the following transformations: `Count the number of occurrences`, `Check if some categories occur row-wise`, and `Sum row-wise`, which allow the creation of a single variable starting from more than one.

   Moreover, we have corrected the transformation "quantitative to categorical" as you suggested. Now the number of categories cannot drop below two.

3. *I tried to replicate the analyses but was unable to do so, as some variables appear to be missing. I would be willing to replicate it again once a revised version is available.*

   We have revised the entire application, including the correct variables to be used in order to perform it. Moreover, the steps of the application are now described in detail in the section *Real Data Application*, in order to facilitate replication. Many details are provided for the harmonization

phases, which may be the most complex to perform through the application.

4. *More generally, the key information necessary for replication should be clearly presented in the main text and then described in greater detail in the appendix. In its current form, the steps are difficult to follow, and replication is challenging. Please revise the manuscript accordingly by integrating more details into the main text*
   We thank the reviewer for highlighting this issue. We agree on the importance of improving reproducibility. To address this point, we have substantially revised the manuscript by moving all essential information required to replicate the application into the main text. In particular:

   - detailed instructions for implementing the application have been added;
   - Tables 1 and 2 now report the variables required for matching;
   - the harmonization procedure is described in detail in the main text.

   Supplementary materials are now used only for additional details and validation (e.g., Tables S3 and S4), while the core steps of the analysis are fully described in the manuscript.

References

D'Orazio, M., Di Zio, M., and Scanu, M. (2006). *Statistical matching: Theory and practice*. John Wiley & Sons.

Rubin, D. B. (1996). Multiple imputation after 18+ years. *Journal of the American statistical Association*, 91(434):473–489.
