# Supplementary material for: ShinyDataMatcher: A user-friendly application for integrating survey data
Source: PLoS One. 2026 Jul 14;21(7):e0353530. doi: 10.1371/journal.pone.0353530 (PMC13367710; doi:10.1371/journal.pone.0353530)
Supplement: Attachment — Submitted filename: letter_reviewers2.pdf [file pone.0353530.s025.pdf]

June 23, 2026

Object: PONE-D-26-04293R1 revised paper submission

Dear Reviewers,

we thank you for carefully reading our paper and highlighting minor points. We hope that this new version improves our work in the direction you indicated. Changes to the previous version have been highlighted in bold in order to facilitate your reading of the revised paper. Please find a point-by-point answer to your comments in what follows (our answers are interlined to your suggestions).

Reply to the Reviewer # 1

Thank you very much for your valuable comments and suggestions. We have carefully considered them and made revisions to address your concerns in this paper version.

1. *Page 10, line 354, "In the field used to select the modality…" perhaps a title for the modality box in the app?*

   The title `Select categories` has been added to the modality box in the app. Moreover, in the manuscript, the text now reads: "In the field used to select the modality (`Select categories`)" (line 336, page 9).

2. *In page 10, line 360, I guess you mean "lower middle school certificate" instead of "secondary school certificate".*

   Thank you for this observation. We have corrected the text by replacing "secondary school certificate" with "lower middle school certificate". The revised wording appears on page 9, line 341.

3. *Page 12, line 467, the predictors in Figure S13 are not the same as specified in the text.*

   To provide greater clarity in the manuscript, we have specified that, for the regression analysis performed on the HBS dataset, the following predictors were selected: `Area, No_tit, Comp, Diploma, Degree, Job, Ret, Housesup, NCOMP`. The results, shown in Figure S13, indicate that the variables with a statistically significant effect on `Z` are `Diploma`, `Degree`, `Ret`, `Job`, `Area`, and `Housesup`. The modified text can be found starting at line 440 on page 11.

Reply to the Reviewer # 2

Thank you very much for your valuable comments and suggestions. We have carefully considered them and made revisions to address your concerns in this paper version.

1. *Studio[…] variables: the category secondary school certificate is missing. Did you mean lower middle school certificate?*

   Thank you for pointing this out. The original wording was incorrect. We have replaced "secondary school certificate" with "lower middle school certificate". This change can now be found on page 9, line 341.

2. *In quantitative to categorical: number of categories can still be negative.*

   Thank you for pointing this out. It has been fixed.
