# Supplementary material for: ShinyDataMatcher: A user-friendly application for integrating survey data
Source: PLoS One. 2026 Jul 14;21(7):e0353530. doi: 10.1371/journal.pone.0353530 (PMC13367710; doi:10.1371/journal.pone.0353530)
Supplement: Attachment — Submitted filename: reviewer1.docx [file pone.0353530.s023.docx]

The revised version improved a lot, and I think it is almost ready for publication. I spotted some minor errors, and I think once these errors are solved, the paper may be published without further revision.

1. Page 10, line 354, “In the field used to select the modality…” perhaps a title for the modality box in the app?
2. In page 10, line 360, I guess you mean “lower middle school certificate” instead of “secondary school certificate”.

3. Page 12, line 467, the predictors in Figure S13 are not the same as specified in the text.
