# Supplementary material for: ShinyDataMatcher: A user-friendly application for integrating survey data
Source: PLoS One. 2026 Jul 14;21(7):e0353530. doi: 10.1371/journal.pone.0353530 (PMC13367710; doi:10.1371/journal.pone.0353530)
Supplement: Peer review history

# Peer review: Decision Letter 0

**DOI:** 10.1371/journal.pone.0353530.r001

**Authors:** Angelo Moretti

4 Mar 2026

PONE-D-26-04293

ShinyDataMatcher: A User-Friendly Application for Integrating Survey Data

PLOS One

Dear Dr. Guastadisegni,

Thank you for submitting your manuscript to PLOS ONE. After careful consideration, we feel that it has merit but does not fully meet PLOS ONE’s publication criteria as it currently stands. Therefore, we invite you to submit a revised version of the manuscript that addresses the points raised during the review process.

Please submit your revised manuscript by Apr 18 2026 11:59PM. If you will need more time than this to complete your revisions, please reply to this message or contact the journal office at plosone@plos.org. When you're ready to submit your revision, log on to https://www.editorialmanager.com/pone/ and select the 'Submissions Needing Revision' folder to locate your manuscript file.

Please include the following items when submitting your revised manuscript:

- A letter that responds to each point raised by the academic editor and reviewer(s). You should upload this letter as a separate file labeled 'Response to Reviewers'.
- A marked-up copy of your manuscript that highlights changes made to the original version. You should upload this as a separate file labeled 'Revised Manuscript with Track Changes'.
- An unmarked version of your revised paper without tracked changes. You should upload this as a separate file labeled 'Manuscript'.

If you would like to make changes to your financial disclosure, please include your updated statement in your cover letter. Guidelines for resubmitting your figure files are available below the reviewer comments at the end of this letter.

If applicable, we recommend that you deposit your laboratory protocols in protocols.io to enhance the reproducibility of your results. Protocols.io assigns your protocol its own identifier (DOI) so that it can be cited independently in the future. For instructions see: https://journals.plos.org/plosone/s/submission-guidelines#loc-laboratory-protocols. Additionally, PLOS ONE offers an option for publishing peer-reviewed Lab Protocol articles, which describe protocols hosted on protocols.io. Read more information on sharing protocols at https://plos.org/protocols?utm_medium=editorial-email&utm_source=authorletters&utm_campaign=protocols.

We look forward to receiving your revised manuscript.

Kind regards,

Angelo Moretti, Ph.D.

Academic Editor

PLOS One

Journal Requirements:

When submitting your revision, we need you to address these additional requirements.

1. Please ensure that your manuscript meets PLOS ONE's style requirements, including those for file naming. The PLOS ONE style templates can be found at

https://journals.plos.org/plosone/s/file?id=wjVg/PLOSOne_formatting_sample_main_body.pdf and

https://journals.plos.org/plosone/s/file?id=ba62/PLOSOne_formatting_sample_title_authors_affiliations.pdf

2. Please note that PLOS One has specific guidelines on code sharing for submissions in which author-generated code underpins the findings in the manuscript. In these cases, we expect all author-generated code to be made available without restrictions upon publication of the work.

Please review our guidelines at https://journals.plos.org/plosone/s/materials-and-software-sharing#loc-sharing-code and ensure that your code is shared in a way that follows best practice and facilitates reproducibility and reuse.

3. Thank you for stating the following financial disclosure:

“This study was funded by the European Union -  NextGenerationEU, in the framework of the “GRINS -Growing Resilient, INclusive and Sustainable project” (PNRR - M4C2 - I1.3 - PE00000018 – CUP J33C22002910001). The views and opinions expressed are solely those of the authors and do not necessarily reflect those of the European Union, nor can the European Union be held responsible for them.”

Please state what role the funders took in the study.  If the funders had no role, please state: "The funders had no role in study design, data collection and analysis, decision to publish, or preparation of the manuscript." If this statement is not correct you must amend it as needed.

Please include this amended Role of Funder statement in your cover letter; we will change the online submission form on your behalf.

4. Please note that funding information should not appear in the Acknowledgments section or other areas of your manuscript. We will only publish funding information present in the Funding Statement section of the online submission form. Please remove any funding-related text from the manuscript.

5. Please ensure that you refer to Figures 1, 3, 4, 5 and 6 in your text as, if accepted, production will need this reference to link the reader to the figure.

6. If the reviewer comments include a recommendation to cite specific previously published works, please review and evaluate these publications to determine whether they are relevant and should be cited. There is no requirement to cite these works unless the editor has indicated otherwise.

**Additional Editor Comments:**

believe this article will be really helpful to statistics matching users. One of the reviewers  (reviewer 2) found issues with the replicability of the examples. I invite you to go over this point carefully before resubmitting it. Other typos in formulas were identified by Reviewer 1, e.g., in the MSE formula in presence of imputation. I think you could make the first part of the paper a bit easier to follow (for example, by focusing a little bit more on the application and app instead of the theory).

[Note: HTML markup is below. Please do not edit.]

Reviewers' comments:

Reviewer's Responses to Questions

**Comments to the Author**

1. Is the manuscript technically sound, and do the data support the conclusions?

The manuscript must describe a technically sound piece of scientific research with data that supports the conclusions. Experiments must have been conducted rigorously, with appropriate controls, replication, and sample sizes. The conclusions must be drawn appropriately based on the data presented.

Reviewer #1: Yes

Reviewer #2: Partly

**********

2. Has the statistical analysis been performed appropriately and rigorously?

Reviewer #1: Yes

Reviewer #2: Yes

**********

3. Have the authors made all data underlying the findings in their manuscript fully available?

The PLOS Data policy requires authors to make all data underlying the findings described in their manuscript fully available without restriction, with rare exception (please refer to the Data Availability Statement in the manuscript PDF file). The data should be provided as part of the manuscript or its supporting information, or deposited to a public repository. For example, in addition to summary statistics, the data points behind means, medians and variance measures should be available. If there are restrictions on publicly sharing data—e.g. participant privacy or use of data from a third party—those must be specified.

Reviewer #1: Yes

Reviewer #2: Yes

**********

4. Is the manuscript presented in an intelligible fashion and written in standard English?

PLOS ONE does not copyedit accepted manuscripts, so the language in submitted articles must be clear, correct, and unambiguous. Any typographical or grammatical errors should be corrected at revision, so please note any specific errors here.

Reviewer #1: Yes

Reviewer #2: Yes

**********

5. Review Comments to the Author

Please use the space provided to explain your answers to the questions above. You may also include additional comments for the author, including concerns about dual publication, research ethics, or publication ethics. (Please upload your review as an attachment if it exceeds 20,000 characters)

Reviewer #1: In general, I like the paper and the app. I think it is relevant and intuitive for people who are interested in statistical matching. I mainly have some suggestions for the construction of the paper. I hope the paper can therefore be more attractive to our readers.

Major:

1. I think it would be better to try to make the theory of statistical matching more concise, and mainly focus on the app itself. After all, the app is the star of this paper and we would not want to exhaust our readers before they really get to the important part. One option may be to leave the technical details of statistical matching to the supplement and only keep the general idea and assumptions of statistical matching in the main text.

2. The workflow has been introduced many times in the section of The workflow of Statistical Matching, ShinyDataMatcher: Overview, and Application. I believe these can be combined or trimmed in a way to overall shorten the paper.

3. As a potential user, I will be interested in how you make the decisions in the application. For example, why you choose these variables for matching, why you harmonize in this way, and why you choose Random Distance Hot Deck.

4. The formula for the variance of multiple imputation is incorrect. It will be better to cite the variance calculation from Rubin (1987) instead of Rubin (1978). Rubin (1996) may also be a nice reference.

Minor:

1. I tried to download the shiny app. The code remotes::install github(fedele-greco/ShinyStatMatcher) does not work for me, but remotes::install github(“fedele-greco/ShinyStatMatcher”) does.

2. In the Application, lots of figures and tables are left in the supplement. I personally find it quite hard to read. Perhaps we can only keep some of the tabs and put them in the main text? I do understand that you may want to keep them as a tutorial. But the app itself is already quite self-explanatory in my opinion.

3. PARENT_- 1_Fact to PARENT_- 9_Fact in table S1 cannot be found in SHIW, and ID, c_relaz_1_-Fact to c_relaz_12_-Fact in table S2 cannot be found in HBS. Therefore, I did not continue trying out the application. I will be happy to do so if there is another round of review.

Reference

Rubin, D. B. (1987). Multiple imputation for nonresponse in surveys. New York, NY.

Rubin, D. B. (1996). Multiple imputation after 18+ years. Journal of the American statistical Association, 91(434), 473-489.

Reviewer #2: The manuscript describes an innovative tool to perform statistical matching; however, in its current form, revisions are required. In particular, it is not possible to replicate the application as presented, and that section should be extensively revised. I therefore recommend major revisions.

Minor Comments

• Page 2/21, line 60: There is a missing quotation mark in the command: remotes::install_github(‘fedele-greco/ShinyStatMatcher’)

• Page 8/21: It is unclear what is meant by “information.” Does this refer only to the raw variables in the dataset that are used to construct the final harmonized variables for matching, to survey design characteristics, or to correlations between variables? Later, _ and _ are denoted as reduced datasets containing candidate variables for matching. This is confusing and should be clarified

• Page 10/21: “List of Available Surveys.” -> Replace the period with a colon for consistency

• Page 13/21: I find the terminology “key variable,” used to refer to unit IDs in the dataset, somewhat confusing. I suggest renaming it to make explicit that it refers to the unit identifier.

• Figure 7: is difficult to read due to too many overlapping arrows and squares

Major comments on the application section

• The selection of relevant “information” remains unclear. Tables S1 and S2 present the list of variables to be selected for matching; however, not all of these variables appear to be available in the dataset. The manuscript should specify explicitly which variables should be selected

• Under Data Wrangling -> Select a transformation (one variable): while multiple options can be selected, the tool does not appear to be designed to operate with multiple transformations simultaneously. In addition, when selecting “quantitative to categorical,” the number of categories can drop below two, even becoming negative. This should be corrected.

• I tried to replicate the analyses but was unable to do so, as some variables appear to be missing. I would be willing to replicate it again once a revised version is available.

• More generally, the key information necessary for replication should be clearly presented in the main text and then described in greater detail in the appendix. In its current form, the steps are difficult to follow, and replication is challenging. Please revise the manuscript accordingly by integrating more details into the main text

**********

6. PLOS authors have the option to publish the peer review history of their article (what does this mean?). If published, this will include your full peer review and any attached files.

If you choose “no”, your identity will remain anonymous but your review may still be made public.

**Do you want your identity to be public for this peer review?**  For information about this choice, including consent withdrawal, please see our Privacy Policy.

Reviewer #1: No

Reviewer #2: No

**********

[NOTE: If reviewer comments were submitted as an attachment file, they will be attached to this email and accessible via the submission site. Please log into your account, locate the manuscript record, and check for the action link "View Attachments". If this link does not appear, there are no attachment files.]

To ensure your figures meet our technical requirements, please review our figure guidelines: https://journals.plos.org/plosone/s/figures

You may also use PLOS’s free figure tool, NAAS, to help you prepare publication quality figures: https://journals.plos.org/plosone/s/figures#loc-tools-for-figure-preparation.

NAAS will assess whether your figures meet our technical requirements by comparing each figure against our figure specifications.

Attachment Submitted filename: review.pdf

# Author comment: Author response to Decision Letter 1

**DOI:** 10.1371/journal.pone.0353530.r002

20 Apr 2026

Reply to the Reviewer # 1

Thank you very much for your valuable comments and suggestions. We have carefully considered them and made revisions to address your concerns in this paper version.

1. I think it would be better to try to make the theory of statistical matching more concise, and mainly focus on the app itself. After all, the app is the star of this paper and we would not want to exhaust our readers before they really get to the important part. One option may be to leave the technical details of statistical matching to the supplement and only keep the general idea and assumptions of statistical matching in the main text.

Following your suggestion, we have substantially streamlined the Statistical Framework and Methods section. The main text now provides only a general overview of statistical matching, while the technical details have been moved to Appendix S1.

In particular, the description of parametric macro methods is now presented in Section S1.1, parametric micro methods in Section S1.2, and non-parametric micro methods in Section S2 of the appendix.

2. The workflow has been introduced many times in the section of The workflow of Statistical Matching, ShinyDataMatcher: Overview, and Application. I believe these can be combined or trimmed in a way to overall shorten the paper

Following your suggestion, we have created a single section that summarizes the information previously presented in The Workflow of Statistical Matching and ShinyDataMatcher: Overview. This section is now titled Statistical matching through ShinyDataMatcher.

However, we have chosen to keep the real data application in a separate section due to its importance, as it can also serve as a tutorial to use the app for users.

3. As a potential user, I will be interested in how you make the decisions in the application. For example, why you choose these variables for matching, why you harmonize in this way, and why you choose Random Distance Hot Deck

Thank you for this important comment. We have clarified the rationale behind the main choices made in the application.

First, the empirical application is designed to replicate the framework of D'Orazio(2006), using more recent data (year 2020). This has now been explicitly stated at the beginning of the Real Data Application section.

Second, the selection of variables is guided by standard practice in statistical matching: users should choose variables that capture comparable information across datasets (e.g., demographic and socio-economic characteristics). In our case, we retained a subset of variables from D'Orazio(2006) to simplify the illustration. This has been clarified in the manuscript.

Third, the harmonization strategy follows the same reference, with additional explanation provided in the revised text. In particular, we clarify why aggregation procedures (e.g., counting occurrences) are used to preserve information at the household level.

Finally, we have clarified the choice of the Random Distance Hot Deck method. This method allows the generation of multiple imputations, which is essential for assessing matching uncertainty. Compared to the standard random hot-deck, it also provides more accurate matches by incorporating a distance metric.

All these clarifications have been added in the relevant sections of the manuscript (sectionReal Data application).

4. The formula for the variance of multiple imputation is incorrect. It will be better to cite the variance calculation from Rubin (1987) instead of Rubin (1978). Rubin (1996) may also be a nice reference

In the subsection Building the SAM matrix, we have corrected the formula of the variance of the multiple imputation (MI) following Rubin(1996). The variance is now specified as:

\begin{equation}

\hat{V}(\bar{Z}) = W + \left(\frac{K+1}{K}\right) B,

\end{equation}

where the formula for the between-imputation variance has been corrected as follows:

\begin{equation}

B = \frac{1}{K-1} \sum_{k=1}^{K} \left(\bar{Z}^{(k)} - \bar{Z}\right)^{2}.

\end{equation}

The results in Table 4 have been modified accordingly.

Minor:

1.I tried to download the shiny app. The code remotes::install github(fedele-greco/ShinyStatMatcher) does not work for me, but remotes::install github(“fedele-greco/ShinyStatMatcher”) does

We have corrected the command line to remotes::install_github("fedele-greco/ShinyStatMatcher") in line 60 of the paper.

2. In the Application, lots of figures and tables are left in the supplement. I personally find it quite hard to read. Perhaps we can only keep some of the tabs and put them in the main text? I do understand that you may want to keep them as a tutorial. But the app itself is already quite self-explanatory in my opinion.

In response to this comment, we have moved all essential information required to perform the real data application into the main text. First, more details on how to actually perform the application, including all the specific features and fields to use, have been added in the section Real Data Application Section. Tables 1 and 2 have been inserted in the subsection Select relevant information for matching and include all the raw variables to be selected for matching. A more detailed version of these tables has been included as supplementary material (Tables S1 and S2). The harmonization phase, described in detail, has also been included in the main text. Tables S3 and S4 have been retained in the supplementary material as a cross-check. Table S5 has been removed, as all the relevant information is now included in the main text. We decided to keep the screenshots in the supplementary material, as they are not essential for actually performing the application.

3. PARENT_1_Fact to PARENT_9_Fact in table S1 cannot be found in SHIW, and ID, c_relaz_1_-Fact to c_relaz_12_-Fact in table S2 cannot be found in HBS. Therefore, I did not continue trying out the application. I will be happy to do so if there is another round of review.

In the previous version of the paper, we made an error in the list of raw variables to be selected in SHIW and HBS, by including some that were not actually useful for the application at hand, as those that you cited in your comment. We have now created the correct tables (Tables 1 and 2), which have been inserted in the main text, along with more detailed versions provided in the supplementary material (Tables S1 and S2).

Reply to the Reviewer # 2

Thank you very much for your valuable comments and suggestions. We have carefully considered them and made revisions to address your concerns in this paper version.

Reply to Minor Comments

1. Page 2/21, line 60: There is a missing quotation mark in the command:remotes::install\_github(‘fedele-greco/ShinyStatMatcher’

We have corrected the command line to remotes::install\_github("fedele-greco/ShinyStatMatcher") in line 60 of the paper.

2. It is unclear what is meant by “information.” Does this refer only to the raw variables in the dataset that are used to construct the final harmonized variables for matching, to survey design characteristics, or to correlations between variables? Later, _ and _ are denoted as reduced datasets containing candidate variables for matching. This is confusing and should be clarified

The term information refers to the raw variables selected from the original datasets that will play a role in the matching procedure, for example as target variables or as variables to be harmonized for matching. The reduced datasets that include these selected raw variables are denoted as $\mathcal{I}_A$ and $\mathcal{I}_B$. We have emphasized this point in the subsection Select Relevant Information from A and B of the section Statistical Matching through ShinyDataMatcher.

The other notation we use refers to the harmonized datasets, denoted as $\mathbf{X}_A$ and $\mathbf{X}_B$. These datasets include candidate matching variables that have been harmonized, as well as the target variables. We have clarified this aspect in the subsection Variables Harmonization of the section Statistical Matching through ShinyDataMatcher.

3. Page 10/21: “List of Available Surveys.” -> Replace the period with a colon for consistency

We have replaced the period with a colon.

4.Page 13/21: I find the terminology “key variable,” used to refer to unit IDs in the dataset, somewhat confusing. I suggest renaming it to make explicit that it refers to the unit identifier.

We have replaced the terminology “key variable” with “ID variable” in both the paper and the app.

5. Figure 7: is difficult to read due to too many overlapping arrows and squares

Following your suggestion, we have split Figure 7 into two figures. Figure 7 now includes only the statistical macro matching methods, while Figure 8 presents only the micro methods.

Reply to Major comments on the application section

1. The selection of relevant “information” remains unclear. Tables S1 and S2 present the list of variables to be selected for matching; however, not all of these variables appear to be available in the dataset. The manuscript should specify explicitly which variables should be selected

We thank the reviewer for this observation. In the previous version, the list of variables included some inconsistencies.

We have now revised the selection of variables and provided a clear and correct list in Tables 1 and 2 in the main text. These tables explicitly indicate the variables required to replicate the application.

Additional details are provided in Tables S1 and S2 in the supplementary material.

2. Under Data Wrangling -> Select a transformation (one variable): while multiple options can be selected, the tool does not appear to be designed to operate with multiple transformations simultaneously. In addition, when selecting “quantitative to categorical,” the number of categories can drop below two, even becoming negative. This should be corrected.

Thank you for pointing this out. The tool is not designed to operate with multiple transformations simultaneously. We have modified the app, and it is now possible to select only one transformation at a time.

If a single variable is selected in the field Select a variable (or a group of variables), the menu Select a transformation (one variable) will appear with the following options: Add as it is, Rename variable, Quantitative to categorical, and Recode a factor.

To visualize the transformation when multiple variables are selected, users need to select more than one variable in the field Select a variable (or a group of variables) to transform in one of the tabs of data wrangling. In this case, a menu Select a transformation (several variables) will appear, offering the following transformations: Count the number of occurrences, Check if some categories occur row-wise, and Sum row-wise, which allow the creation of a single variable starting from more than one.

Moreover, we have corrected the transformation “quantitative to categorical” as you suggested. Now the number of categories cannot drop below two.

3. I tried to replicate the analyses but was unable to do so, as some variables appear to be missing. I would be willing to replicate it again once a revised version is available.

We have revised the entire application, including the correct variables to be used in order to perform it. Moreover, the steps of the application are now described in detail in the section \textit{Real Data Application}, in order to facilitate replication. Many details are provided for the harmonization phases, which may be the most complex to perform through the application.

4. More generally, the key information necessary for replication should be clearly presented in the main text and then described in greater detail in the appendix. In its current form, the steps are difficult to follow, and replication is challenging. Please revise the manuscript accordingly by integrating more details into the main text

We thank the reviewer for highlighting this issue. We agree on the importance of improving reproducibility.

To address this point, we have substantially revised the manuscript by moving all essential information required to replicate the application into the main text. In particular:

- detailed instructions for implementing the application have been added;

- Tables 1 and 2 now report the variables required for matching;

- the harmonization procedure is described in detail in the main text.

Supplementary materials are now used only for additional details and validation (e.g., Tables S3 and S4), while the core steps of the analysis are fully described in the manuscript.

Attachment Submitted filename: response reviewers.pdf

# Peer review: Decision Letter 1

**DOI:** 10.1371/journal.pone.0353530.r003

**Authors:** Angelo Moretti, Angelo Moretti

19 Jun 2026

PONE-D-26-04293R1

ShinyDataMatcher: A User-Friendly Application for Integrating Survey Data

PLOS One

Dear Dr. Guastadisegni,

Thank you for submitting your manuscript to PLOS ONE. After careful consideration, we feel that it has merit but does not fully meet PLOS ONE’s publication criteria as it currently stands. Therefore, we invite you to submit a revised version of the manuscript that addresses the points raised during the review process.

The reviewers recommended minor revisions which you can find in attachment.

Please submit your revised manuscript by Aug 03 2026 11:59PM. If you will need more time than this to complete your revisions, please reply to this message or contact the journal office at plosone@plos.org. When you're ready to submit your revision, log on to https://www.editorialmanager.com/pone/ and select the 'Submissions Needing Revision' folder to locate your manuscript file.

Please include the following items when submitting your revised manuscript:

- A letter that responds to each point raised by the academic editor and reviewer(s). You should upload this letter as a separate file labeled 'Response to Reviewers'.
- A marked-up copy of your manuscript that highlights changes made to the original version. You should upload this as a separate file labeled 'Revised Manuscript with Track Changes'.
- An unmarked version of your revised paper without tracked changes. You should upload this as a separate file labeled 'Manuscript'.

If you would like to make changes to your financial disclosure, please include your updated statement in your cover letter. Guidelines for resubmitting your figure files are available below the reviewer comments at the end of this letter.

If applicable, we recommend that you deposit your laboratory protocols in protocols.io to enhance the reproducibility of your results. Protocols.io assigns your protocol its own identifier (DOI) so that it can be cited independently in the future. For instructions see: https://journals.plos.org/plosone/s/submission-guidelines#loc-laboratory-protocols. Additionally, PLOS ONE offers an option for publishing peer-reviewed Lab Protocol articles, which describe protocols hosted on protocols.io. Read more information on sharing protocols at https://plos.org/protocols?utm_medium=editorial-email&utm_source=authorletters&utm_campaign=protocols.

As the corresponding author, your ORCID iD is verified in the submission system and will appear in the published article. PLOS supports the use of ORCID, and we encourage all coauthors to register for an ORCID iD and use it as well. Please encourage your coauthors to verify their ORCID iD within the submission system before final acceptance, as unverified ORCID iDs will not appear in the published article. *Only*  the individual author can complete the verification step; PLOS staff *cannot*  verify ORCID iDs on behalf of authors.

We look forward to receiving your revised manuscript.

Kind regards,

Angelo Moretti, Ph.D.

Academic Editor

PLOS One

Journal Requirements:

1. If the reviewer comments include a recommendation to cite specific previously published works, please review and evaluate these publications to determine whether they are relevant and should be cited. There is no requirement to cite these works unless the editor has indicated otherwise.

2. Please review your reference list to ensure that it is complete and correct. If you have cited papers that have been retracted, please include the rationale for doing so in the manuscript text, or remove these references and replace them with relevant current references. Any changes to the reference list should be mentioned in the rebuttal letter that accompanies your revised manuscript. If you need to cite a retracted article, indicate the article’s retracted status in the References list and also include a citation and full reference for the retraction notice.

[Note: HTML markup is below. Please do not edit.]

Reviewers' comments:

Reviewer's Responses to Questions

**Comments to the Author**

1. If the authors have adequately addressed your comments raised in a previous round of review and you feel that this manuscript is now acceptable for publication, you may indicate that here to bypass the “Comments to the Author” section, enter your conflict of interest statement in the “Confidential to Editor” section, and submit your "Accept" recommendation.

Reviewer #1: All comments have been addressed

Reviewer #2: All comments have been addressed

**********

2. Is the manuscript technically sound, and do the data support the conclusions?

The manuscript must describe a technically sound piece of scientific research with data that supports the conclusions. Experiments must have been conducted rigorously, with appropriate controls, replication, and sample sizes. The conclusions must be drawn appropriately based on the data presented.

Reviewer #1: Yes

Reviewer #2: Yes

**********

3. Has the statistical analysis been performed appropriately and rigorously?

Reviewer #1: Yes

Reviewer #2: Yes

**********

4. Have the authors made all data underlying the findings in their manuscript fully available?

The PLOS Data policy requires authors to make all data underlying the findings described in their manuscript fully available without restriction, with rare exception (please refer to the Data Availability Statement in the manuscript PDF file). The data should be provided as part of the manuscript or its supporting information, or deposited to a public repository. For example, in addition to summary statistics, the data points behind means, medians and variance measures should be available. If there are restrictions on publicly sharing data—e.g. participant privacy or use of data from a third party—those must be specified.

Reviewer #1: Yes

Reviewer #2: Yes

**********

5. Is the manuscript presented in an intelligible fashion and written in standard English?

PLOS ONE does not copyedit accepted manuscripts, so the language in submitted articles must be clear, correct, and unambiguous. Any typographical or grammatical errors should be corrected at revision, so please note any specific errors here.

Reviewer #1: Yes

Reviewer #2: Yes

**********

6. Review Comments to the Author

Please use the space provided to explain your answers to the questions above. You may also include additional comments for the author, including concerns about dual publication, research ethics, or publication ethics. (Please upload your review as an attachment if it exceeds 20,000 characters)

Reviewer #1: The revised version improved a lot, and I think it is almost ready for publication. I spotted some minor errors, and I think once these errors are solved, the paper may be published without further revision.

1. Page 10, line 354, “In the field used to select the modality…” perhaps a title for the modality box in the app?

2. In page 10, line 360, I guess you mean “lower middle school certificate” instead of “secondary school certificate”.

3. Page 12, line 467, the predictors in Figure S13 are not the same as specified in the text.

Reviewer #2: I thank the authors for their revision. My previous comments have been addressed.

I have only a few minor comments.

When replicating the analyses:

- Studio[…] variables: the category secondary school certificate is missing. Did you mean lower middle school certificate?

- In quantitative to categorical: number of categories can still be negative.

**********

7. PLOS authors have the option to publish the peer review history of their article (what does this mean?). If published, this will include your full peer review and any attached files.

If you choose “no”, your identity will remain anonymous but your review may still be made public.

**Do you want your identity to be public for this peer review?**  For information about this choice, including consent withdrawal, please see our Privacy Policy.

Reviewer #1: **Yes:** An-Chiao Liu

Reviewer #2: No

**********

[NOTE: If reviewer comments were submitted as an attachment file, they will be attached to this email and accessible via the submission site. Please log into your account, locate the manuscript record, and check for the action link "View Attachments". If this link does not appear, there are no attachment files.]

To ensure your figures meet our technical requirements, please review our figure guidelines: https://journals.plos.org/plosone/s/figures

You may also use PLOS’s free figure tool, NAAS, to help you prepare publication quality figures: https://journals.plos.org/plosone/s/figures#loc-tools-for-figure-preparation.

NAAS will assess whether your figures meet our technical requirements by comparing each figure against our figure specifications.

Attachment Submitted filename: reviewer1.docx

# Author comment: Author response to Decision Letter 2

**DOI:** 10.1371/journal.pone.0353530.r004

23 Jun 2026

Reply to the Reviewer # 1

Thank you very much for your valuable comments and suggestions. We have carefully considered them and made revisions to address your concerns in this paper version.

1. Page 10, line 354, “In the field used to select the modality…” perhaps a title for the modality box in the app?

The title "Select categories" has been added to the modality box in the app. Moreover, in the manuscript, the text now reads: "In the field used to select the modality ("Select categories")" (line 336, page 9).

2. In page 10, line 360, I guess you mean “lower middle school certificate” instead of “secondary school certificate”.

Thank you for this observation. We have corrected the text by replacing “secondary school certificate” with “lower middle school certificate”. The revised wording appears on page 9, line 341.

3. Page 12, line 467, the predictors in Figure S13 are not the same as specified in the text.

To provide greater clarity in the manuscript, we have specified that, for the regression analysis performed on the HBS dataset, the following predictors were selected: Area, No_tit, Comp, Diploma, Degree, Job, Ret, Housesup, NCOMP. The results, shown in Figure~S13, indicate that the variables with a statistically significant effect on Z are Diploma, Degree, Ret, Job, Area, and Housesup. The modified text can be found starting at line 440 on page 11.

Reply to the Reviewer # 2

Thank you very much for your valuable comments and suggestions. We have carefully considered them and made revisions to address your concerns in this paper version.

1. Studio[…] variables: the category secondary school certificate is missing. Did you mean lower middle school certificate?

Thank you for pointing this out. The original wording was incorrect. We have replaced “secondary school certificate” with “lower middle school certificate”. This change can now be found on page 9, line 341.

2. In quantitative to categorical: number of categories can still be negative.

Thank you for pointing this out. It has been fixed.

Attachment Submitted filename: letter_reviewers2.pdf

# Editor report: Decision Letter 2

**DOI:** 10.1371/journal.pone.0353530.r005

**Authors:** Angelo Moretti, Angelo Moretti, Angelo Moretti

24 Jun 2026

ShinyDataMatcher: A User-Friendly Application for Integrating Survey Data

PONE-D-26-04293R2

Dear Dr. Guastadisegni,

We’re pleased to inform you that your manuscript has been judged scientifically suitable for publication and will be formally accepted for publication once it meets all outstanding technical requirements.

Within one week, you’ll receive an e-mail detailing the required amendments. When these have been addressed, you’ll receive a formal acceptance letter and your manuscript will be scheduled for publication.

An invoice will be generated when your article is formally accepted. Please note, if your institution has a publishing partnership with PLOS and your article meets the relevant criteria, all or part of your publication costs will be covered. Please make sure your user information is up-to-date by logging into Editorial Manager at Editorial Manager® and clicking the ‘Update My Information' link at the top of the page. For questions related to billing, please contact billing support.

If your institution or institutions have a press office, please notify them about your upcoming paper to help maximize its impact. If they’ll be preparing press materials, please inform our press team as soon as possible -- no later than 48 hours after receiving the formal acceptance. Your manuscript will remain under strict press embargo until 2 pm Eastern Time on the date of publication. For more information, please contact onepress@plos.org.

Kind regards,

Angelo Moretti, Ph.D.

Academic Editor

PLOS One

Additional Editor Comments (optional):

Reviewers' comments:

# Editor report: Acceptance letter

**DOI:** 10.1371/journal.pone.0353530.r006

**Authors:** Angelo Moretti, Angelo Moretti, Angelo Moretti

PONE-D-26-04293R2

PLOS One

Dear Dr. Guastadisegni,

I'm pleased to inform you that your manuscript has been deemed suitable for publication in PLOS One. Congratulations! Your manuscript is now being handed over to our production team.

At this stage, our production department will prepare your paper for publication. This includes ensuring the following:

* All references, tables, and figures are properly cited

* All relevant supporting information is included in the manuscript submission,

* There are no issues that prevent the paper from being properly typeset

You will receive further instructions from the production team, including instructions on how to review your proof when it is ready. Please keep in mind that we are working through a large volume of accepted articles, so please give us a few days to review your paper and let you know the next and final steps.

Lastly, if your institution or institutions have a press office, please let them know about your upcoming paper now to help maximize its impact. If they'll be preparing press materials, please inform our press team within the next 48 hours. Your manuscript will remain under strict press embargo until 2 pm Eastern Time on the date of publication. For more information, please contact onepress@plos.org.

You will receive an invoice from PLOS for your publication fee after your manuscript has reached the completed accept phase. If you receive an email requesting payment before acceptance or for any other service, this may be a phishing scheme. Learn how to identify phishing emails and protect your accounts at https://explore.plos.org/phishing.

If we can help with anything else, please email us at customercare@plos.org.

Thank you for submitting your work to PLOS ONE and supporting open access.

Kind regards,

PLOS ONE Editorial Office Staff

on behalf of

Dr. Angelo Moretti

Academic Editor

PLOS One
